# Supplementary material for: The physical activity health paradox and risk factors for cardiovascular disease: A cross-sectional compositional data analysis in the Copenhagen City Heart Study
Source: PLoS One. 2022 Apr 21;17(4):e0267427. doi: 10.1371/journal.pone.0267427 (PMC9022831; doi:10.1371/journal.pone.0267427)
Supplement: S1 Table — (PDF) [file pone.0267427.s001.pdf]

Supporting Information Table S1

Table S1. Overview of questions and responses used in the present study from the questionnaire in the fifth examination of the Copenhagen City Heart Study

**Question**	**Possible responses**
Level of education	
What education have you completed since you left municipal primary and lower secondary school?	No education<br>Short education (≤3 years with books)<br>Vocational or similar education (1-3 years)<br>Higher education (≥3 years, e.g., teacher, nurse or similar)<br>University education
Occupation/employment	
What occupation have you had for the longest time, since you completed your education/school years?	Self-employed<br>Skilled/trained<br>Unskilled<br>White-collar/non-manual worker<br>Housewife/ house husband<br>Student<br>Unemployed/retired
Household income	
What was your total household income before tax last year?	<100 000 DKK<br>100 000 – 200 000 DKK<br>200 000 – 400 000 DKK<br>400 000 – 600 000 DKK<br>600 000 – 800 000 DKK<br>>800 000 DKK
Civil status	
At the present moment, are you?	Married/cohabiting<br>Unmarried<br>Separated/divorced<br>Widow/widower
Self-rated fitness compared to peers	
How do you rate your fitness compared to your peers?	Same<br>Better<br>Worse
Lifestyle/Smoking status	
Do you smoke?<br>If no, have you previously smoked?	Yes<br>No
Self-reported general health	
How do you think your health is all in all?	Excellent<br>Very good<br>Good<br>Less good<br>Poor
All questions have been freely translated from Danish to English for the purpose of this overview only.<br>DKK, Danish kroner	
