# Supplementary material for: The physical activity health paradox and risk factors for cardiovascular disease: A cross-sectional compositional data analysis in the Copenhagen City Heart Study
Source: PLoS One. 2022 Apr 21;17(4):e0267427. doi: 10.1371/journal.pone.0267427 (PMC9022831; doi:10.1371/journal.pone.0267427)
Supplement: S2 Table — (PDF) [file pone.0267427.s002.pdf]

Supporting Information Table S2

**Table S2.** Overview of derived variables used in the present study, based on data from the questionnaire and physical examination in the fifth examination of the Copenhagen City Heart Study	
**Variable and question**	**Possible responses and definition**
Covariates	
Sex	
	Women<br>Men<br>Derived from civil registration number<br>Number of missing values: 0
Age	
	Age at date of physical examination, derived from date of birth.<br>Number of missing values: 0
Number of years of education	
"*How many years have you gone to school? (primary or secondary school, high school or similar) (max. 14 years)*"	Participants filled out the number of years in free text.<br>Number of missing values: 0
Average number of alcohol units per week	
Participants were asked about the average number of beer(s) (33 cl), white wine (12.5 cl), red wine (12.5 cl), liqueur (8 cl) or spirits (4 cl) per week.	Participants filled out the number of units in free text.<br>The average number of units was calculated by taking the sum of the reported alcohol consumption.<br>In case of all values missing, the participant was assigned a missing value.<br>Number of missing values: 79 among adults, 38 among older adults
Smoking status	
Study participants were categorised as smokers, previous smokers and non-smokers based on the questions *"Do you smoke?"* and *"If no, have you previously smoked?"* with response categories *"Yes"* and *"No"*.	Those answering "Yes" to "*Do you smoke?*" were categorised as smokers.<br>Those answering "No" to "*Do you smoke?*", and "*If no, have you previously smoked?*" were categorised as non-smokers.<br>Those answering "Yes" to "*If no, have you previously smoked?*" were categorised as previous smokers.<br>Number of missing values: 15 among adults, 6 among older adults
Self-reported use of prescribed medication	
"Do you daily or almost daily take:"	Participants indicated their answer ("Yes" or "No") for each listed class of medication.<br>Those answering "Yes" to anticoagulants, antihypertensives, heart medicine (i.e., for heart failure, etc.), diuretics, cholesterol lowering medication, insulin, other medication for diabetes, or antidepressants or sedatives, were considered to use prescribed medication.<br>Those not answering yes, and "No" to any of the items were classified as not taking prescribed medication.<br>If all items were missing, the participant was assigned a missing value.<br>Number of missing values: 0

**Table S2.** (Continued)	
**Variable and question**	**Possible responses and definition**
Variables for descriptive purposes	
BMI	
BMI was derived from measured height and weight (calculated as weight in kilograms divided by height in meters squared)	BMI was categorised into (according to WHO classification):<br>*Underweight* (<18.5 kg/m^2)<br>N*ormal weight* (18.5-<25.0 kg/m^2)<br>O*verweight* (25.0-<30.0 kg/m^2)<br>*Obese* (≥30 kg/m^2<br>Number of missing values: 0
Blood pressure classification	
Blood pressure was measured as part of the physical examination.	Blood pressure was categorised into:<br>*Normal* (systolic: <140 mmHg and diastolic: <90 mmHg; i.e., including high normal),<br>*Grade 1 hypertension* (systolic: 140-≤159 mmHg or diastolic: 90-≤99 mmHg),<br>*Grade 2 hypertension* (systolic: 160-≤179 mmHg or diastolic: 100-≤109 mmHg),<br>*Grade 3 hypertension* (systolic: ≥180 mmHg or diastolic: ≥110 mmHg).<br>Number of missing values: 0
Waist circumference (categorised)	
Waist circumference was measured as part of the physical examination.	WC was categorised into:<br>>88 cm for women<br>>94 cm for men.<br>Number of missing values: 0
Household income	
"*What was your total household income before tax last year?*"	Response categories:<br><100 000 DKK<br>100 000 to 200 000 DKK<br>200 000 to 400 000 DKK<br>400 000 to 600 000 DKK<br>600 000 to 800 000 DKK<br>>800 000 DKK<br>Response categories grouped into:<br>Low (<200 000 DKK),<br>Moderate (200 000 to 600 000 DKK)<br>High (>600 000 DKK)<br>Number of missing values: 8 among adults, 11 among older adults
Self-rated fitness compared to peers	
"*How do you rate your fitness compared to your peers?*"	Response categories:<br>Same<br>Better<br>Worse<br>Number of missing values: 2 among adults, 1 among older adults

**Table S2.** (Continued)	
**Variable and question**	**Possible responses and definition**
Variables for descriptive purposes	
Self-rated general health	
"How do you think your health is all in all?"	Response categories:<br>Excellent<br>Very good<br>Good<br>Less good<br>Poor<br>Response categories grouped into:<br>Excellent and very good<br>Good<br>Less good and poor<br>Number of missing values:
All questions have been freely translated from Danish to English for the purpose of this overview only.<br>BMI, body mass index<br>WC, waist circumference<br>DKK, Danish krone	
