# Supplementary material for: The physical activity health paradox and risk factors for cardiovascular disease: A cross-sectional compositional data analysis in the Copenhagen City Heart Study
Source: PLoS One. 2022 Apr 21;17(4):e0267427. doi: 10.1371/journal.pone.0267427 (PMC9022831; doi:10.1371/journal.pone.0267427)
Supplement: S3 Table — (PDF) [file pone.0267427.s003.pdf]

Supporting Information Table S3

Table S3. Variation matrix of parts in physical activity composition among 652 adults participating in the fifth examination of the Copenhagen City Heart Study

Physical behaviour	*Sedentary W*	*Standing W*	*Moving W*	*Walking W*	*HIPA W*	*Sedentary L*	*Standing L*	*Moving L*	*Walking L*	*HIPA L*	*Time in bed*
Sedentary W	0	1.301	1.459	0.997	2.099	0.529	0.732	0.649	0.630	1.623	0.457
Standing W		0	0.266	0.329	1.784	0.612	0.519	0.599	0.644	1.718	0.489
Moving W			0	0.213	1.624	0.706	0.748	0.675	0.764	1.923	0.626
Walking W				0	1.514	0.453	0.566	0.517	0.482	1.675	0.398
HIPA W					0	1.730	1.819	1.774	1.829	1.951	1.626
Sedentary L						0	0.260	0.228	0.201	1.319	0.082
Standing L							0	0.088	0.162	1.177	0.171
Moving L								0	0.112	1.131	0.161
Walking L									0	1.178	0.166
HIPA L										0	1.194
Time in bed											0

W/L, work and leisure
Small values indicate a high co-dependency between two parts of the physical behaviour composition, and vice versa for large values.
HIPA-types, sum of climbing stairs (up/down), running, cycling and rowing.
Green indicate values in the range >0 to ≤0.5; orange indicate values in the range >0.5 to ≤1.0; red indicate values >1.0.
