# Supplementary material for: The physical activity health paradox and risk factors for cardiovascular disease: A cross-sectional compositional data analysis in the Copenhagen City Heart Study
Source: PLoS One. 2022 Apr 21;17(4):e0267427. doi: 10.1371/journal.pone.0267427 (PMC9022831; doi:10.1371/journal.pone.0267427)
Supplement: S4 Table — (PDF) [file pone.0267427.s004.pdf]

Supporting Information Table S4

Table S4. Comparison of characteristics of 1367 non-eligible and 652 eligible participants from the fifth examination of the Copenhagen City Heart Study (Denmark)

***N* = 2019**	**Non-eligible participants *n* = 1367**	**Eligible participants *n* = 652**	***P***
**Characteristics**	**n (%) [95% CI] Median [95% CI]**	**n (%) [95% CI] Median [95% CI]**	
Accelerometer wear time			**<0.001**
Median minutes/day	1435 [1432-1436]	1425 [1423-1427]	
Number of valid days of measurement			**<0.001**
Median number of days	6 [6-6]	6 [6-6]	
Working hours			0.092
Median minutes/day	450 [440-465]	453 [450-461]	
Number of workdays			**<0.001**
Median number of days	0 [0-0]	4 [4-4]	
Sex distribution			0.951
Women	789 (58%) [55-60]	378 (58%) [54-62]	
Men	578 (42%) [40-45]	274 (42%) [38-46]	
Age			**<0.001**
Median years	66 [66-67]	49 [47-50]	
Years of education			**<0.001**
Median years	11 [11-11]	13 [12-13]	
Level of education			**<0.001**
No [further] education	176 (13%) [11-15]	47 (7%) [6-10]	
Short education (up to 3 years)	156 (12%) [10-13]	44 (7%) [5-9]	
Vocational or comparable education (1-3 years)	395 (29%) [27-32]	105 (16%) [14-19]	
Higher education (≥3 years)	335 (25%) [22-27]	176 (27%) [24-31]	
University education	300 (22%) [20-24]	279 (43%) [39-47]	
Household income			**<0.001**
Low (<200 000 DKK)	365 (27%) [25-30]	69 (11%) [9-13]	
Middle (200 000-600 000 DKK)	661 (50%) [47-52]	238 (37%) [33-41]	
High (≥600 000 DKK)	310 (23%) [21-26]	337 (52%) [49-56]	
Smoking status			**<0.001**
Non-smoker	496 (37%) [34-40]	295 (46%) [42-50]	
Previous smoker	596 (44%) [42-47]	253 (40%) [36-43]	
Current smoker	254 (19%) [17-21]	91 (14%) [12-17]	
Average weekly number of units of alcohol per week			0.090
Median units/week	7 [7-7]	6 [6-7]	

**Table S4.** (Continued)			
**Characteristics**	**n (%) [95% CI]<br>Median [95% CI]**	**n (%) [95% CI]<br>Median [95% CI]**	***P***
Use of prescribed medication			**<0.001**
No	598 (44%) [41-47]	603 (93%) [90-94]	
Yes	761 (56%) [53-59]	49 (8%) [6-10]	
Self-rated fitness compared to peers			0.325
Same	629 (46%) [44-49]	324 (50%) [46-54]	
Better	513 (38%) [35-40]	232 (36%) [32-39]	
Worse	218 (16%) [14-18]	95 (15%) [12-18]	
Self-reported general health			**<0.001**
Excellent Very good	498 (37%) [34-39]	328 (51%) [47-55]	
Good	593 (44%) [41-46]	256 (40%) [36-43]	
Less good or Poor	269 (20%) [18-22]	64 (10%) [8-12]	
Systolic blood pressure			**<0.001**
Median (mm Hg)	139 [137 -140]	128 [126-129]	
Diastolic blood pressure			0.418
Median (mm Hg)	78 [77-78]	77 [76-78]	
Blood pressure classification			**<0.001**
Normal	699 (52%) [49-54]	486 (76%) [71-78]	
Grade 1 hypertension	513 (38%) [35-41]	147 (23%) [20-26]	
Grade 2 or 3 hypertension	139 (10%) [9-12]	19 (3%) [2-5]	
Waist circumference			**<0.001**
Median (cm)	89 [89-90]	83 [82-84]	
WC (women >80 cm, men >94 cm)			**<0.001**
Under cut-point	572 (42%) [40-45]	413 (63%) [60-67]	
Above cut-point	787 (58%) [55-61]	239 (37%) [33-40]	
BMI, WHO classification			**<0.001**
Normal (incl. underweight)	596 (44%) [41-46]	393 (60.3%) [56.5-64]	
Overweight	547 (40%) [38-43]	203 (31.1%) [27.7-34.8]	
Obese	221 (16%) [14-18]	56 (8.6%) [6.7-11]	
Low-density lipoprotein cholesterol			
Median (mmol/L)	3.0 [2.9-3.0]	3.0 [2.9-3.1]	0.110

The non-eligible includes 191 individuals with missing information on whether they agreed to wear accelerometers or not.
N/n, number of observations
P, p-value from Mann-Whitney U test and Pearson's Chi-squared test
CI, confidence interval, CIs were calculated using the Wilson score method for proportions and the normal approximation method for medians.
DKK, Danish krone
Blood pressure classification is based on the 2013 European Society of Hypertension/European Society of Cardiology guidelines for the management of arterial hypertension (the normal category includes high normal).
WC, waist circumference
BMI, body mass index; WHO's classification: underweight, <18.5 kg/m^2; normal, 18.5-<25.0 kg/m^2; overweight, 25.0-<30.0 kg/m^2; obese, ≥30 kg/m^2. The underweight category was merged with the normal weight category because of low number of underweight individuals. The normal BMI-category includes 13 and 7 underweight individuals in the non-eligible and eligible participants, respectively.
