# Supplementary material for: The physical activity health paradox and risk factors for cardiovascular disease: A cross-sectional compositional data analysis in the Copenhagen City Heart Study
Source: PLoS One. 2022 Apr 21;17(4):e0267427. doi: 10.1371/journal.pone.0267427 (PMC9022831; doi:10.1371/journal.pone.0267427)
Supplement: S1 File — (PDF) [file pone.0267427.s005.pdf]

Supporting Information File S1: Linear regression models

Systolic blood pressure

Table A. Model output from crude systolic blood pressure-model based on 652 observations

Variable	Estimate	Standard error	95% CI	
			Lower	Upper
Intercept	126.613	5.238	116.327	136.899
ilr1	1.010	1.160	-1.267	3.287
ilr2	1.659	1.827	-1.929	5.246
ilr3	1.750	2.114	-2.402	5.902
ilr4	1.453	1.936	-2.349	5.255
ilr5	1.538	0.631	0.299	2.776
ilr6	12.219	2.659	6.998	17.440
ilr7	-1.147	3.238	-7.506	5.212
ilr8	3.400	3.322	-3.122	9.923
ilr9	2.482	2.444	-2.317	7.282
ilr10	3.186	2.496	-1.715	8.087

CI, confidence interval
ilr1-ilr10, iso-metric log-ratio coordinates representing the transformed physical behaviour composition
ilr1 represent sedentary behaviour relative to the geometric mean of the remaining behaviours in the daily physical behaviour composition

Table B. Model output from adjusted systolic blood pressure-model based on 583 observations

Variable	Estimate	Standard error	95% CI	
			Lower	Upper
Intercept	96.074	8.811	78.767	113.382
ilr1	0.332	1.071	-1.771	2.435
ilr2	0.175	1.639	-3.045	3.395
ilr3	-0.957	1.954	-4.795	2.880
ilr4	2.726	1.823	-0.854	6.306
ilr5	0.728	0.596	-0.443	1.899
ilr6	9.548	2.543	4.553	14.543
ilr7	2.883	3.002	-3.013	8.778
ilr8	-4.358	3.122	-10.489	1.774
ilr9	1.966	2.268	-2.488	6.420
ilr10	2.322	2.364	-2.322	6.966
Age	0.459	0.055	0.351	0.567
Sex<br>Men vs. Women	7.811	1.330	5.199	10.423
Level of education	-0.178	0.460	-1.082	0.725
Smoking status<br>Previous smoker vs. Never smoker	1.177	1.341	-1.457	3.810
Smoking status<br>Smoker vs. Never smoker	2.136	1.972	-1.737	6.009
Average units of alcohol/week	0.201	0.079	0.046	0.356
Use of prescribed medicine<br>Yes vs. No	-2.389	2.347	-6.998	2.221

CI, confidence interval
ilr1-ilr10, iso-metric log-ratio coordinates representing the transformed physical behaviour composition
ilr1 represent sedentary behaviour relative to the geometric mean of the remaining behaviours in the daily physical behaviour composition

Model validation

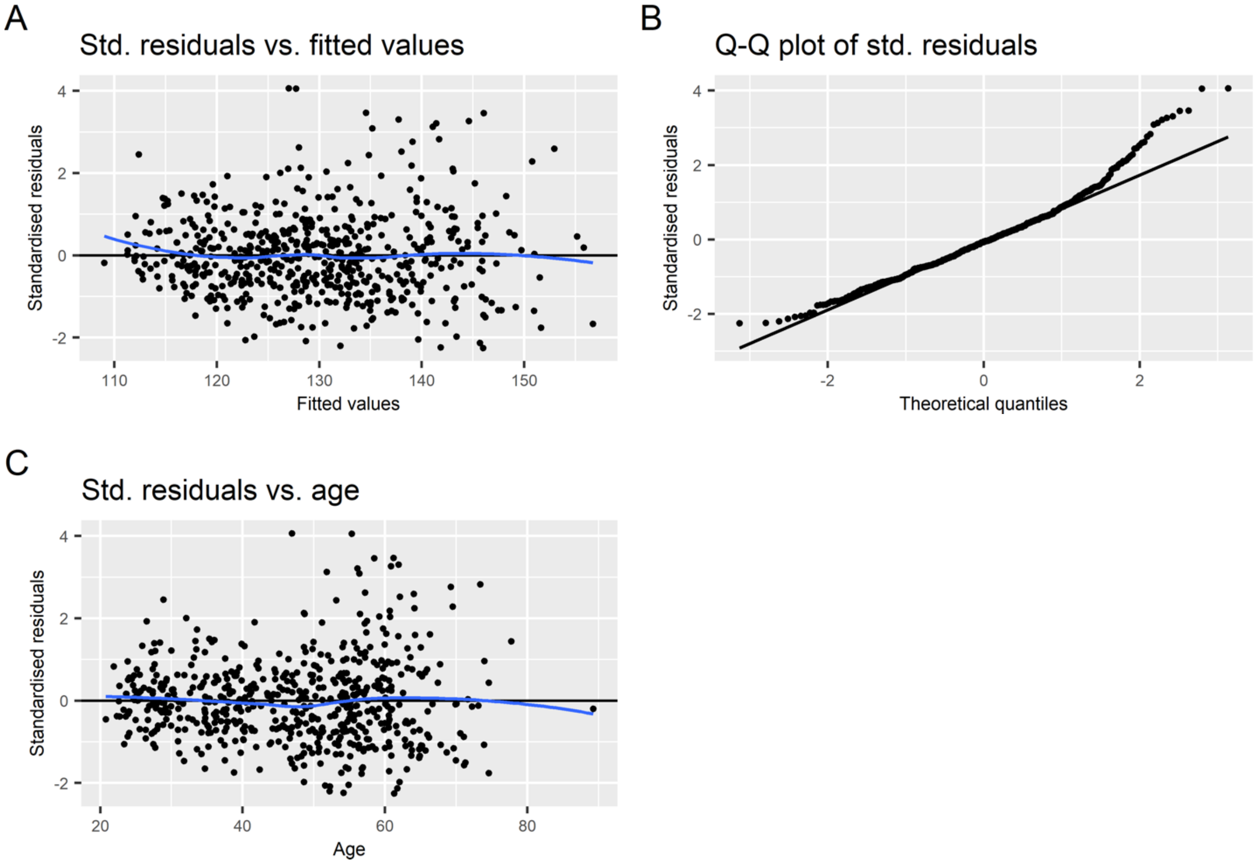

Figure A.

Waist circumference

Table C. Model output from crude waist circumference-model based on 652 observations

Variable	Estimate	Standard error	95% CI	
			Lower	Upper
Intercept	75.002	3.472	68.184	81.819
ilr1	1.175	0.769	-0.335	2.684
ilr2	0.636	1.211	-1.741	3.014
ilr3	0.060	1.401	-2.692	2.811
ilr4	2.452	1.283	-0.068	4.972
ilr5	-0.058	0.418	-0.879	0.763
ilr6	7.824	1.762	4.364	11.284
ilr7	-1.533	2.146	-5.748	2.681
ilr8	1.649	2.202	-2.674	5.972
ilr9	2.638	1.620	-0.543	5.819
ilr10	0.520	1.654	-2.728	3.768

CI, confidence interval
ilr1-ilr10, iso-metric log-ratio coordinates representing the transformed physical behaviour composition
ilr1 represent sedentary behaviour relative to the geometric mean of the remaining behaviours in the daily physical behaviour composition

Table D. Model output from adjusted waist circumference-model based on 583 observations

Variable	Estimate	Standard error	95% CI	
			Lower	Upper
Intercept	63.878	5.698	52.687	75.069
ilr1	0.623	0.692	-0.737	1.983
ilr2	0.653	1.060	-1.429	2.735
ilr3	-1.458	1.263	-3.939	1.024
ilr4	2.124	1.179	-0.190	4.439
ilr5	-0.060	0.386	-0.817	0.698
ilr6	4.508	1.644	1.278	7.738
ilr7	1.309	1.941	-2.503	5.121
ilr8	-2.358	2.018	-6.322	1.607
ilr9	2.977	1.466	0.097	5.857
ilr10	-0.042	1.529	-3.045	2.961
Age	0.189	0.035	0.119	0.259
Sex<br>Men vs. Women	8.162	0.860	6.474	9.851
Level of education	-0.215	0.297	-0.799	0.369
Smoking status<br>Previous smoker vs. Never smoker	0.680	0.867	-1.023	2.383
Smoking status<br>Smoker vs. Never smoker	-0.357	1.275	-2.861	2.147
Average units of alcohol/week	0.098	0.051	-0.002	0.199
Use of prescribed medicine<br>Yes vs. No	2.930	1.518	-0.051	5.910

CI, confidence interval
ilr1-ilr10, iso-metric log-ratio coordinates representing the transformed physical behaviour composition
ilr1 represent sedentary behaviour relative to the geometric mean of the remaining behaviours in the daily physical behaviour composition

Model validation

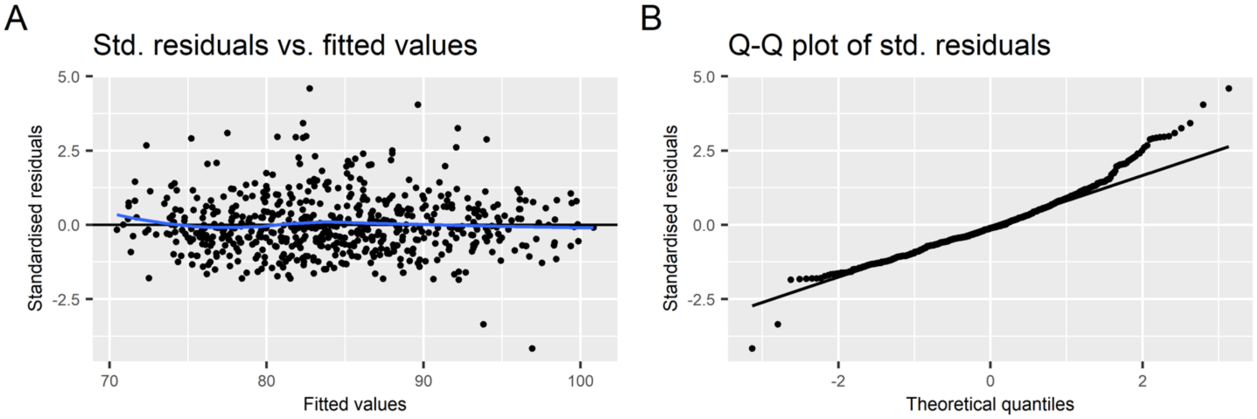

Figure B.

Low-density lipoprotein cholesterol

Table E. Model output from crude low-density lipoprotein-model based on 652 observations

Variable	Estimate	Standard error	95% CI	
			Lower	Upper
Intercept	2.670	0.280	2.120	3.219
ilr1	-0.022	0.062	-0.144	0.099
ilr2	-0.120	0.098	-0.312	0.072
ilr3	0.009	0.113	-0.213	0.230
ilr4	0.088	0.103	-0.115	0.291
ilr5	-0.006	0.034	-0.072	0.061
ilr6	0.290	0.142	0.011	0.569
ilr7	-0.209	0.173	-0.549	0.131
ilr8	0.068	0.177	-0.281	0.416
ilr9	0.239	0.131	-0.017	0.496
ilr10	-0.116	0.133	-0.378	0.146

CI, confidence interval
ilr1-ilr10, iso-metric log-ratio coordinates representing the transformed physical behaviour composition
ilr1 represent sedentary behaviour relative to the geometric mean of the remaining behaviours in the daily physical behaviour composition

Table F. Model output from adjusted low-density lipoprotein-model based on 583 observations

Variable	Estimate	Standard error	95% CI	
			Lower	Upper
Intercept	1.724	0.522	0.698	2.750
ilr1	-0.043	0.063	-0.168	0.081
ilr2	-0.209	0.097	-0.400	-0.018
ilr3	-0.037	0.116	-0.264	0.191
ilr4	0.112	0.108	-0.100	0.324
ilr5	-0.037	0.035	-0.107	0.032
ilr6	0.212	0.151	-0.084	0.508
ilr7	-0.019	0.178	-0.369	0.330
ilr8	-0.295	0.185	-0.659	0.068
ilr9	0.286	0.134	0.022	0.550
ilr10	-0.084	0.140	-0.359	0.191
Age	0.018	0.003	0.012	0.025
Sex<br>Men vs. Women	0.190	0.079	0.035	0.345
Level of education	-0.013	0.027	-0.067	0.040
Smoking status<br>Previous smoker vs. Never smoker	0.122	0.079	-0.034	0.278
Smoking status<br>Smoker vs. Never smoker	0.066	0.117	-0.164	0.295
Average units of alcohol/week	0.004	0.005	-0.005	0.013
Use of prescribed medicine<br>Yes vs. No	0.068	0.139	-0.206	0.341

CI, confidence interval
ilr1-ilr10, iso-metric log-ratio coordinates representing the transformed physical behaviour composition
ilr1 represent sedentary behaviour relative to the geometric mean of the remaining behaviours in the daily physical behaviour composition

Model validation

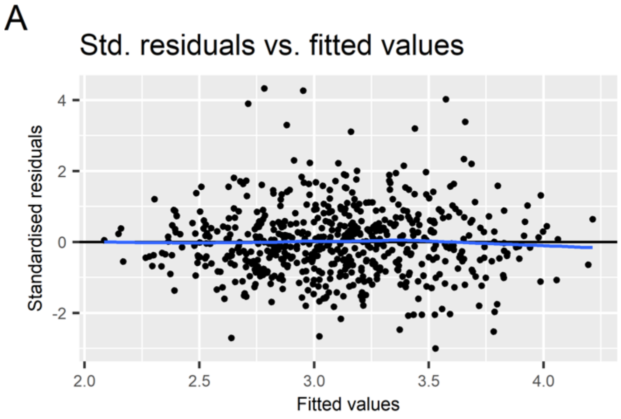

Figure C.
