# Supplementary material for: The physical activity health paradox and risk factors for cardiovascular disease: A cross-sectional compositional data analysis in the Copenhagen City Heart Study
Source: PLoS One. 2022 Apr 21;17(4):e0267427. doi: 10.1371/journal.pone.0267427 (PMC9022831; doi:10.1371/journal.pone.0267427)
Supplement: S2 File — (PDF) [file pone.0267427.s006.pdf]

Supporting Information File S2: Time reallocations

Systolic blood pressure

Table A. Estimated crude differences in systolic blood pressure given time reallocations between sedentary behaviour and walking and sedentary behaviour and high intensity physical activity during leisure and work among 652 adults in the fifth examination of the Copenhagen City Heart Study

Reallocation (min)	Work Estimated difference in mm Hg w. 95% CI			Leisure Estimated difference in mm Hg w. 95% CI		
Sedentary behaviour – walking	**Estimate**	**Lower**	**Upper**	**Estimate**	**Lower**	**Upper**
-50 (sedentary behaviour → walking)				-2.636	-5.776	0.503
-40				-2.143	-4.779	0.493
-30	0.444	-2.213	3.102	-1.639	-3.725	0.446
-20	0.337	-1.604	2.278	-1.119	-2.595	0.356
-10	0.194	-0.887	1.276	-0.577	-1.366	0.213
0 (reference composition)	0.000	0.000	0.000	0.000	0.000	0.000
10	-0.284	-1.755	1.187	0.627	-0.306	1.560
20	-0.763	-4.568	3.042	1.333	-0.748	3.414
30	-2.142	-12.294	8.010	2.174	-1.410	5.757
40				3.283	-2.497	9.063
50 (walking → sedentary behaviour)				5.191	-4.818	15.200
Sedentary behaviour – HIPA						
-10 (sedentary behaviour → HIPA)				-1.991	-2.859	-1.123
-8				-1.670	-2.403	-0.936
-6				-1.320	-1.904	-0.735
-4				-0.933	-1.349	-0.516
-2	0.439	-0.258	1.136	-0.498	-0.723	-0.274
-1	0.252	-0.147	0.651	-0.258	-0.375	-0.142
0 (reference composition)	0.000	0.000	0.000	0.000	0.000	0.000
1	-0.387	-0.997	0.222	0.281	0.153	0.409
2	-1.248	-3.202	0.707	0.588	0.318	0.858
4				1.313	0.704	1.922
6				2.270	1.204	3.336
8				3.710	1.942	5.478
10 (sedentary behaviour → HIPA)				6.885	3.534	10.236

652 observations were included in the crude model.
CI, confidence interval
mm Hg, mm of mercury
HIPA, high-intensity physical activity (sum of climbing stairs [up/down], running, cycling, and rowing)
Reallocations were done relative to the reference composition (i.e., geometric mean): 372.7 and 234.2 min sedentary behaviour, 127.0 and 74.6 min standing, 49.1 and 22.5 min moving, 56.8 and 32.5 min walking, and 10.9 and 2.5 min HIPA, during leisure and work, respectively, and 457.3 min in bed.

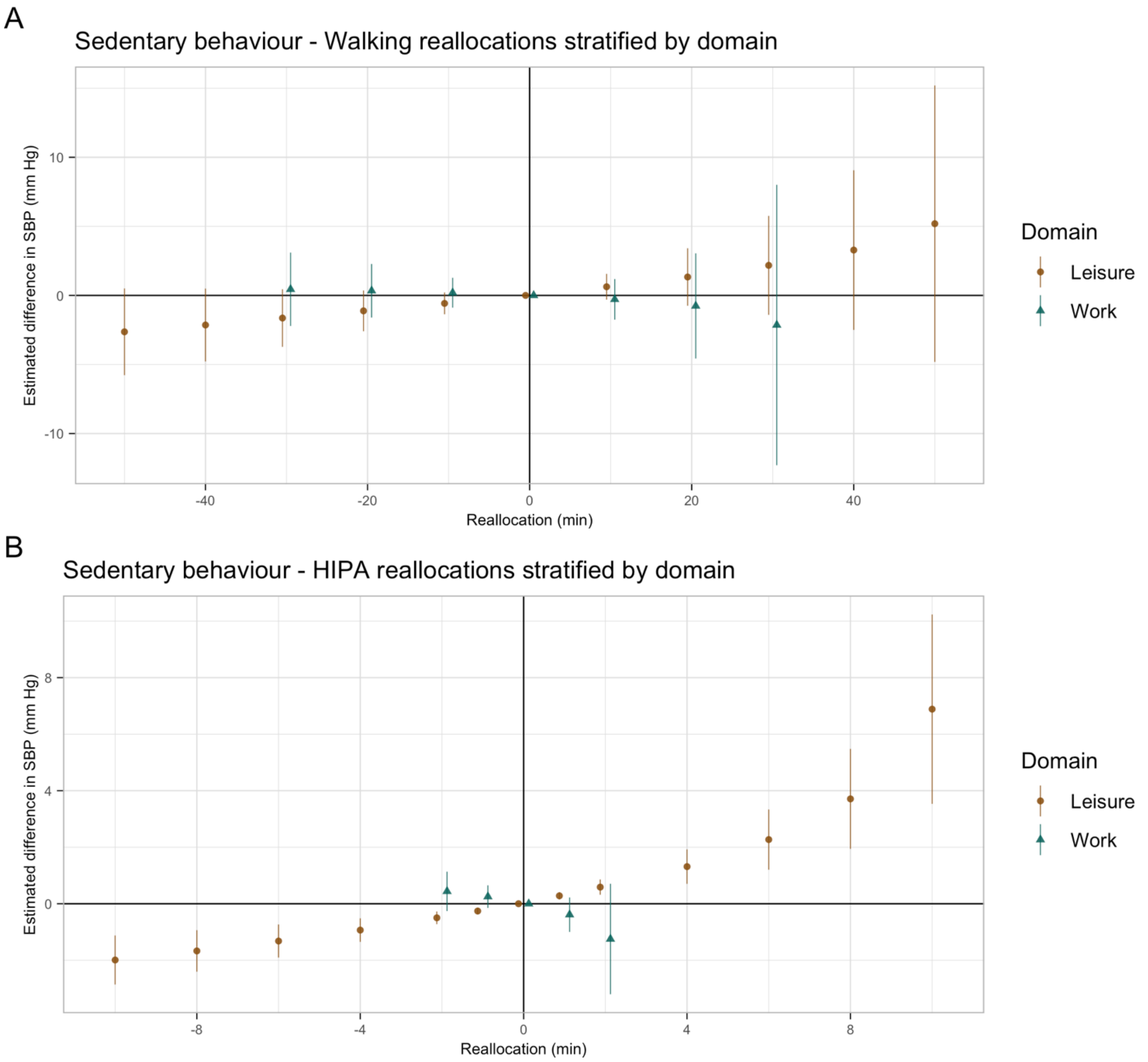

Figure A. Illustration of crude estimated differences in systolic blood pressure (mm Hg) given the reallocation of time between **A)** sedentary behaviour and walking, and **B)** sedentary behaviour and HIPA during work and leisure among 652 adults. A negative reallocated value reflects the pairwise reallocation of time from sedentary behaviour to walking or HIPA, while a positive value reflects the increase in sedentary behaviour at the cost of walking or HIPA. Vertical lines correspond to the 95% confidence intervals. HIPA is high-intensity physical activity (i.e., sum of climbing stairs, running, cycling, and rowing).

Table B. Estimated adjusted differences in systolic blood pressure given time reallocations between sedentary behaviour and walking and sedentary behaviour and high intensity physical activity during leisure and work among 652 adults in the fifth examination of the Copenhagen City Heart Study

**Reallocation (min)**	**Work Estimated difference in mm Hg w. 95% CI**			**Leisure Estimated difference in mm Hg w. 95% CI**		
Sedentary behaviour – walking	**Estimate**	**Lower**	**Upper**	**Estimate**	**Lower**	**Upper**
-50 (sedentary behaviour → walking)				-1.157	-4.008	1.694
-40				-0.910	-3.303	1.483
-30	1.665	-0.830	4.160	-0.671	-2.564	1.222
-20	1.225	-0.598	3.047	-0.439	-1.779	0.900
-10	0.687	-0.328	1.702	-0.216	-0.932	0.500
0 (reference composition)	0.000	0.000	0.000	0.000	0.000	0.000
10	-0.947	-2.328	0.434	0.208	-0.639	1.055
20	-2.470	-6.042	1.103	0.407	-1.481	2.295
30	-6.656	-16.187	2.875	0.597	-2.655	3.848
40				0.771	-4.473	6.016
50 (walking → sedentary behaviour)				0.915	-8.167	9.997
Sedentary behaviour – HIPA						
-10 (sedentary behaviour → HIPA)				-0.685	-1.537	0.167
-8				-0.569	-1.288	0.151
-6				-0.444	-1.017	0.129
-4				-0.310	-0.718	0.098
-2	0.219	-0.440	0.877	-0.164	-0.383	0.056
-1	0.125	-0.252	0.503	-0.084	-0.199	0.030
0 (reference composition)	0.000	0.000	0.000	0.000	0.000	0.000
1	-0.193	-0.769	0.384	0.090	-0.035	0.216
2	-0.619	-2.466	1.228	0.188	-0.077	0.452
4				0.412	-0.184	1.008
6				0.698	-0.344	1.740
8				1.114	-0.613	2.840
10 (sedentary behaviour → HIPA)				1.995	-1.275	5.264

Due to missing values in some covariates, 583 observations were included in the adjusted model.
CI, confidence interval
mm Hg, mm of mercury
HIPA, high-intensity physical activity (sum of climbing stairs [up/down], running, cycling, and rowing)
Reallocations were done relative to the reference composition (i.e., geometric mean): 372.7 and 234.2 min sedentary behaviour, 127.0 and 74.6 min standing, 49.1 and 22.5 min moving, 56.8 and 32.5 min walking, and 10.9 and 2.5 min HIPA, during leisure and work, respectively, and 457.3 min in bed.

Waist circumference

Table C. Estimated crude differences in waist circumference given time reallocations between sedentary behaviour and walking and sedentary behaviour and high intensity physical activity during leisure and work among 652 adults in the fifth examination of the Copenhagen City Heart Study

**Reallocation (min)**	**Work Estimated difference in cm w. 95% CI**			**Leisure Estimated difference in cm w. 95% CI**		
Sedentary behaviour – walking	**Estimate**	**Lower**	**Upper**	**Estimate**	**Lower**	**Upper**
-50 (sedentary behaviour → walking)				-0.901	-2.982	1.180
-40				-0.707	-2.454	1.040
-30	1.225	-0.537	2.986	-0.520	-1.902	0.862
-20	0.911	-0.376	2.197	-0.340	-1.318	0.638
-10	0.516	-0.200	1.233	-0.166	-0.689	0.357
0 (reference composition)	0.000	0.000	0.000	0.000	0.000	0.000
10	-0.728	-1.703	0.247	0.159	-0.460	0.777
20	-1.922	-4.444	0.601	0.309	-1.070	1.688
30	-5.265	-11.994	1.463	0.448	-1.927	2.823
40				0.570	-3.261	4.401
50 (walking → sedentary behaviour)				0.650	-5.984	7.284
Sedentary behaviour – HIPA						
-10 (sedentary behaviour → HIPA)				-1.991	-2.566	-1.415
-8				-1.675	-2.161	-1.188
-6				-1.327	-1.715	-0.940
-4				-0.941	-1.217	-0.665
-2	-0.347	-0.809	0.115	-0.504	-0.653	-0.356
-1	-0.198	-0.463	0.067	-0.262	-0.340	-0.185
0 (reference composition)	0.000	0.000	0.000	0.000	0.000	0.000
1	0.300	-0.104	0.704	0.286	0.201	0.370
2	0.957	-0.339	2.252	0.600	0.421	0.779
4				1.344	0.940	1.748
6				2.333	1.627	3.040
8				3.834	2.662	5.006
10 (sedentary behaviour → HIPA)				7.169	4.949	9.390

652 observations were included in the crude model.
CI, confidence interval
HIPA, high-intensity physical activity (sum of climbing stairs [up/down], running, cycling, and rowing)
Reallocations were done relative to the reference composition (i.e., geometric mean): 372.7 and 234.2 min sedentary behaviour, 127.0 and 74.6 min standing, 49.1 and 22.5 min moving, 56.8 and 32.5 min walking, and 10.9 and 2.5 min HIPA, during leisure and work, respectively, and 457.3 min in bed.

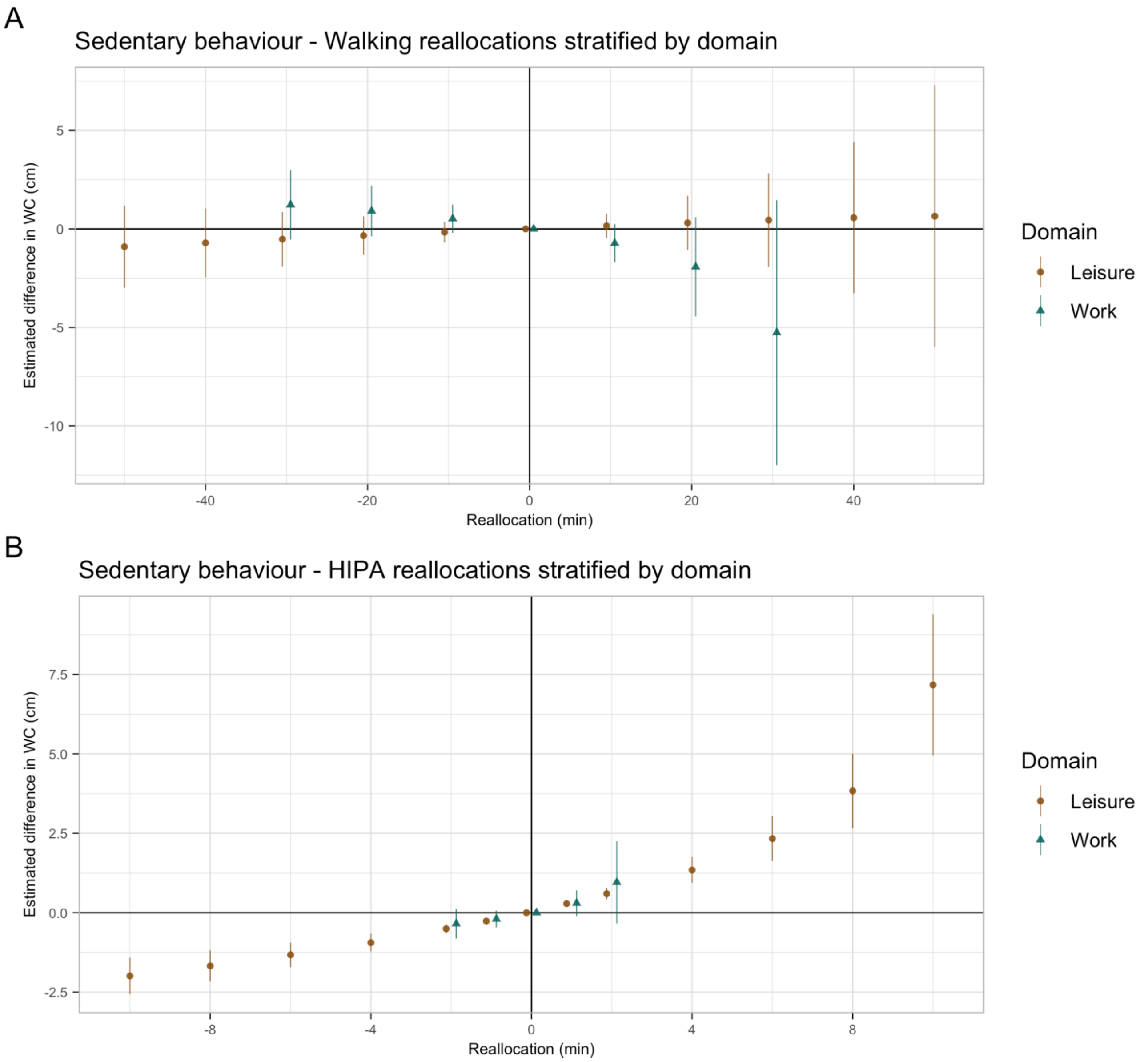

Figure B. Illustration of crude estimated differences in waist circumference (cm) given the reallocation of time between **A)** sedentary behaviour and walking, and **B)** sedentary behaviour and HIPA during work and leisure among 652 adults. A negative reallocated value reflects the pairwise reallocation of time from sedentary behaviour to walking or HIPA, while a positive value reflects the increase in sedentary behaviour at the cost of walking or HIPA. Vertical lines correspond to the 95% confidence intervals. HIPA is high-intensity physical activity (i.e., sum of climbing stairs, running, cycling, and rowing).

Table D. Estimated adjusted differences in waist circumference given time reallocations between sedentary behaviour and walking and sedentary behaviour and high intensity physical activity during leisure and work among 652 adults in the fifth examination of the Copenhagen City Heart Study

**Reallocation (min)**	**Work Estimated difference in cm w. 95% CI**			**Leisure Estimated difference in cm w. 95% CI**		
Sedentary behaviour – walking	**Estimate**	**Lower**	**Upper**	**Estimate**	**Lower**	**Upper**
-50 (sedentary behaviour → walking)				0.555	-1.289	2.398
-40				0.500	-1.048	2.047
-30	1.246	-0.367	2.859	0.422	-0.802	1.646
-20	0.921	-0.258	2.099	0.318	-0.548	1.184
-10	0.519	-0.138	1.175	0.180	-0.283	0.644
0 (reference composition)	0.000	0.000	0.000	0.000	0.000	0.000
10	-0.722	-1.615	0.171	-0.239	-0.786	0.309
20	-1.891	-4.201	0.419	-0.563	-1.784	0.658
30	-5.131	-11.294	1.032	-1.025	-3.127	1.078
40				-1.753	-5.144	1.638
50 (walking → sedentary behaviour)				-3.256	-9.129	2.616
Sedentary behaviour – HIPA						
-10 (sedentary behaviour → HIPA)				-1.352	-1.903	-0.801
-8				-1.138	-1.603	-0.673
-6				-0.903	-1.273	-0.532
-4				-0.641	-0.904	-0.377
-2	-0.181	-0.606	0.245	-0.344	-0.486	-0.201
-1	-0.103	-0.347	0.141	-0.179	-0.253	-0.105
0 (reference composition)	0.000	0.000	0.000	0.000	0.000	0.000
1	0.156	-0.216	0.529	0.195	0.114	0.276
2	0.498	-0.696	1.693	0.409	0.238	0.580
4				0.918	0.533	1.303
6				1.595	0.922	2.269
8				2.625	1.508	3.742
10 (sedentary behaviour → HIPA)				4.918	2.804	7.032

Due to missing values in some covariates, 583 observations were included in the adjusted model.
CI, confidence interval
HIPA, high-intensity physical activity (sum of climbing stairs [up/down], running, cycling, and rowing)
Reallocations were done relative to the reference composition (i.e., geometric mean): 372.7 and 234.2 min sedentary behaviour, 127.0 and 74.6 min standing, 49.1 and 22.5 min moving, 56.8 and 32.5 min walking, and 10.9 and 2.5 min HIPA, during leisure and work, respectively, and 457.3 min in bed.

Low-density lipoprotein cholesterol

Table E. Estimated crude differences in low-density lipoprotein cholesterol given time reallocations between sedentary behaviour and walking and sedentary behaviour and high intensity physical activity during leisure and work among 652 adults in the fifth examination of the Copenhagen City Heart Study

**Reallocation (min)**	**Work Estimated difference in mmol/L w. 95% CI**			**Leisure Estimated difference in mmol/L w. 95% CI**		
Sedentary behaviour – walking	**Estimate**	**Lower**	**Upper**	**Estimate**	**Lower**	**Upper**
-50 (sedentary behaviour → walking)				0.070	-0.097	0.238
-40				0.062	-0.079	0.202
-30	0.066	-0.076	0.208	0.051	-0.061	0.162
-20	0.048	-0.056	0.152	0.037	-0.042	0.116
-10	0.027	-0.031	0.085	0.021	-0.021	0.063
0 (reference composition)	0.000	0.000	0.000	0.000	0.000	0.000
10	-0.036	-0.115	0.042	-0.026	-0.076	0.024
20	-0.094	-0.297	0.110	-0.061	-0.172	0.050
30	-0.249	-0.792	0.293	-0.109	-0.300	0.083
40				-0.183	-0.492	0.126
50 (walking → sedentary behaviour)				-0.332	-0.867	0.202
Sedentary behaviour – HIPA						
-10 (sedentary behaviour → HIPA)				-0.139	-0.186	-0.093
-8				-0.117	-0.157	-0.078
-6				-0.093	-0.124	-0.062
-4				-0.066	-0.089	-0.044
-2	-0.002	-0.039	0.036	-0.036	-0.048	-0.024
-1	-0.001	-0.022	0.020	-0.019	-0.025	-0.012
0 (reference composition)	0.000	0.000	0.000	0.000	0.000	0.000
1	0.002	-0.031	0.034	0.020	0.013	0.027
2	0.005	-0.099	0.110	0.043	0.028	0.057
4				0.096	0.063	0.128
6				0.167	0.110	0.224
8				0.275	0.180	0.369
10 (sedentary behaviour → HIPA)				0.517	0.338	0.696

652 observations were included in the crude model.
CI, confidence interval
mmol/L, mmol per litre
HIPA, high-intensity physical activity (sum of climbing stairs [up/down], running, cycling, and rowing)
Reallocations were done relative to the reference composition (i.e., geometric mean): 372.7 and 234.2 min sedentary behaviour, 127.0 and 74.6 min standing, 49.1 and 22.5 min moving, 56.8 and 32.5 min walking, and 10.9 and 2.5 min HIPA, during leisure and work, respectively, and 457.3 min in bed.

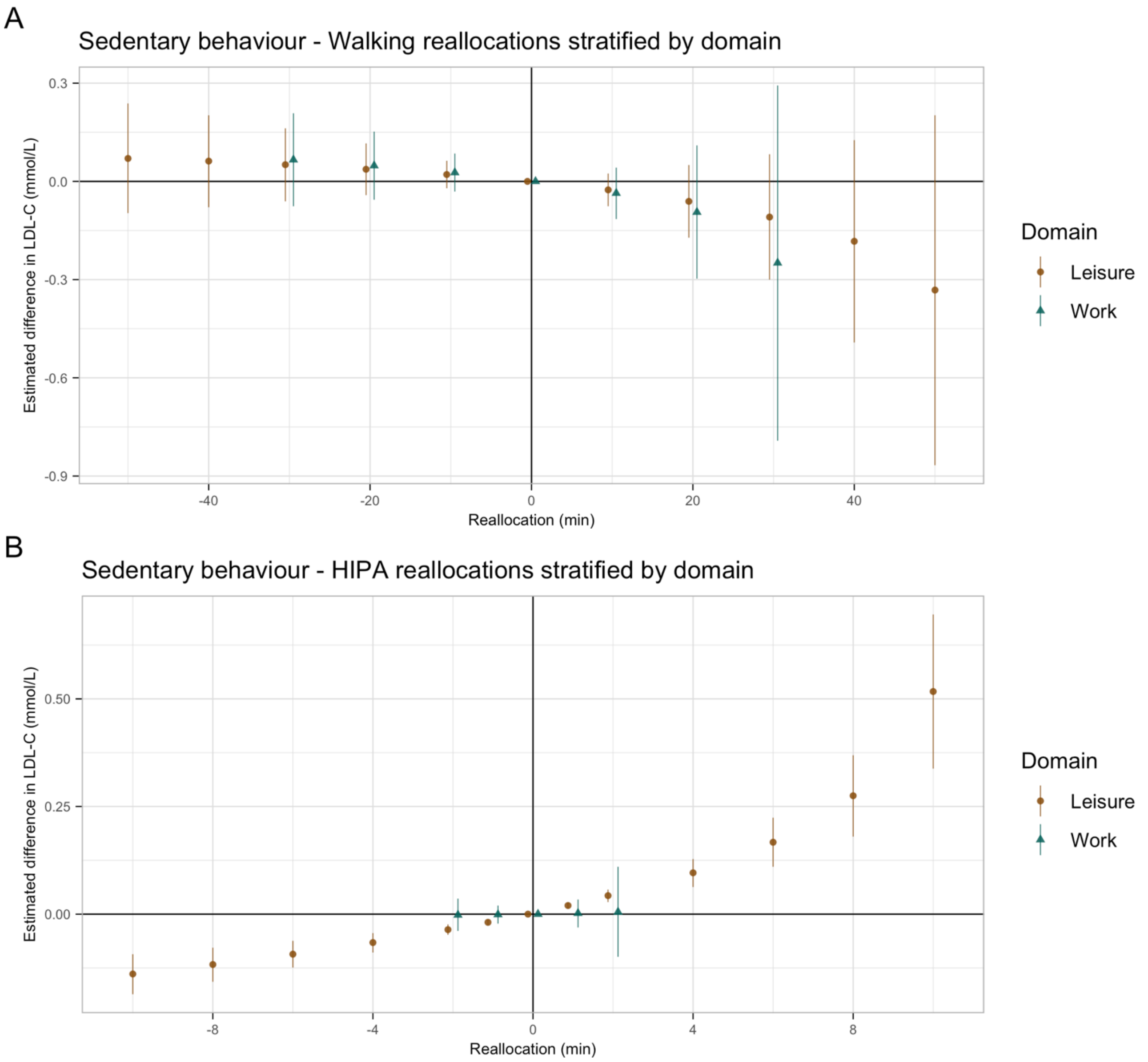

Figure C. Illustration of crude estimated differences in low-density lipoprotein (mmol/L) given the reallocation of time between **A)** sedentary behaviour and walking, and **B)** sedentary behaviour and HIPA during work and leisure among 652 adults. A negative reallocated value reflects the pairwise reallocation of time from sedentary behaviour to walking or HIPA, while a positive value reflects the increase in sedentary behaviour at the cost of walking or HIPA. Vertical lines correspond to the 95% confidence intervals. HIPA is high-intensity physical activity (i.e., sum of climbing stairs, running, cycling, and rowing).

Table F. Estimated adjusted differences in low-density lipoprotein cholesterol given time reallocations between sedentary behaviour and walking and sedentary behaviour and high intensity physical activity during leisure and work among 652 adults in the fifth examination of the Copenhagen City Heart Study

**Reallocation (min)**	**Work Estimated difference in mmol/L w. 95% CI**			**Leisure Estimated difference in mmol/L w. 95% CI**		
Sedentary behaviour – walking	**Estimate**	**Lower**	**Upper**	**Estimate**	**Lower**	**Upper**
-50 (sedentary behaviour → walking)				0.162	-0.007	0.331
-40				0.139	-0.003	0.280
-30	0.094	-0.054	0.242	0.112	-0.001	0.224
-20	0.069	-0.039	0.177	0.080	0.001	0.160
-10	0.038	-0.022	0.098	0.044	0.001	0.086
0 (reference composition)	0.000	0.000	0.000	0.000	0.000	0.000
10	-0.052	-0.133	0.030	-0.054	-0.104	-0.003
20	-0.133	-0.345	0.079	-0.121	-0.233	-0.010
30	-0.352	-0.917	0.213	-0.213	-0.406	-0.020
40				-0.351	-0.661	-0.040
50 (walking → sedentary behaviour)				-0.622	-1.160	-0.084
Sedentary behaviour – HIPA						
-10 (sedentary behaviour → HIPA)				-0.074	-0.124	-0.023
-8				-0.062	-0.105	-0.019
-6				-0.049	-0.083	-0.015
-4				-0.035	-0.059	-0.011
-2	-0.011	-0.050	0.028	-0.019	-0.032	-0.006
-1	-0.006	-0.029	0.016	-0.010	-0.017	-0.003
0 (reference composition)	0.000	0.000	0.000	0.000	0.000	0.000
1	0.010	-0.024	0.044	0.011	0.003	0.018
2	0.032	-0.078	0.141	0.022	0.007	0.038
4				0.050	0.015	0.085
6				0.087	0.025	0.149
8				0.143	0.040	0.245
10 (sedentary behaviour → HIPA)				0.268	0.074	0.461

Due to missing values in some covariates, 583 observations were included in the adjusted model.
CI, confidence interval
mm Hg, mm of mercury
HIPA, high-intensity physical activity (sum of climbing stairs [up/down], running, cycling, and rowing)
Reallocations were done relative to the reference composition (i.e., geometric mean): 372.7 and 234.2 min sedentary behaviour, 127.0 and 74.6 min standing, 49.1 and 22.5 min moving, 56.8 and 32.5 min walking, and 10.9 and 2.5 min HIPA, during leisure and work, respectively, and 457.3 min in bed.
