# Supplementary material for: The physical activity health paradox and risk factors for cardiovascular disease: A cross-sectional compositional data analysis in the Copenhagen City Heart Study
Source: PLoS One. 2022 Apr 21;17(4):e0267427. doi: 10.1371/journal.pone.0267427 (PMC9022831; doi:10.1371/journal.pone.0267427)
Supplement: S3 File — (PDF) [file pone.0267427.s007.pdf]

Supporting Information File S3: Sensitivity analyses

No exclusions based on medication use

In the following sensitivity analyses, we did not exclude individuals taking antihypertensives, diuretics, or cholesterol lowering drugs.

Systolic blood pressure

Table A. Estimated adjusted differences in systolic blood pressure given time reallocations between sedentary behaviour and walking and sedentary behaviour and high intensity physical activity during leisure and work among 804 adults in the fifth examination of the Copenhagen City Heart Study

**Reallocation (min)**	**Work Estimated difference in mm Hg w. 95% CI**			**Leisure Estimated difference in mm Hg w. 95% CI**		
Sedentary behaviour – walking	**Estimate**	**Lower**	**Upper**	**Estimate**	**Lower**	**Upper**
-50 (sedentary behaviour → walking)				-1.545	-4.242	1.151
-40				-1.232	-3.498	1.033
-30	2.262	0.102	4.421	-0.923	-2.717	0.871
-20	1.665	0.091	3.240	-0.616	-1.887	0.655
-10	0.935	0.059	1.810	-0.309	-0.990	0.372
0 (reference composition)	0.000	0.000	0.000	0.000	0.000	0.000
10	-1.284	-2.464	-0.104	0.315	-0.493	1.124
20	-3.315	-6.330	-0.300	0.643	-1.167	2.454
30	-8.344	-15.831	-0.857	0.997	-2.140	4.135
40				1.410	-3.717	6.537
50 (walking → sedentary behaviour)				2.029	-7.269	11.327
Sedentary behaviour – HIPA						
-8 (sedentary behaviour → HIPA)				-0.324	-1.057	0.410
-6				-0.249	-0.836	0.338
-4				-0.171	-0.591	0.250
-2	0.192	-0.446	0.829	-0.088	-0.317	0.140
-1	0.110	-0.255	0.475	-0.045	-0.165	0.074
0 (reference composition)	0.000	0.000	0.000	0.000	0.000	0.000
1	-0.170	-0.726	0.386	0.047	-0.085	0.180
2	-0.544	-2.309	1.220	0.097	-0.184	0.379
4				0.209	-0.438	0.857
6				0.349	-0.826	1.525
8 (HIPA → sedentary behaviour)				0.564	-1.570	2.698

Due to missing values in some covariates, 707 observations were included in the adjusted model.
CI, confidence interval
mm Hg, mm of mercury
HIPA, high-intensity physical activity (sum of climbing stairs [up/down], running, cycling, and rowing)
Reallocations were done relative to the reference composition (i.e., geometric mean): 380.7 and 227.9 min sedentary behaviour, 125.4 and 76.2 min standing, 48.5 and 23.4 min moving, 55.3 and 33.4 min walking, and 9.7 and 2.5 min HIPA, during leisure and work, respectively, and 457.1 min in bed.

Waist circumference

Table B. Estimated adjusted differences in waist circumference given time reallocations between sedentary behaviour and walking and sedentary behaviour and high intensity physical activity during leisure and work among 804 adults in the fifth examination of the Copenhagen City Heart Study

**Reallocation (min)**	**Work Estimated difference in mm Hg w. 95% CI**			**Leisure Estimated difference in mm Hg w. 95% CI**		
Sedentary behaviour – walking	**Estimate**	**Lower**	**Upper**	**Estimate**	**Lower**	**Upper**
-50 (sedentary behaviour → walking)				0.826	-0.864	2.516
-40				0.731	-0.689	2.151
-30	1.163	-0.191	2.516	0.609	-0.516	1.733
-20	0.856	-0.131	1.843	0.452	-0.344	1.249
-10	0.480	-0.068	1.029	0.254	-0.173	0.680
0 (reference composition)	0.000	0.000	0.000	0.000	0.000	0.000
10	-0.660	-1.399	0.080	-0.330	-0.836	0.177
20	-1.704	-3.593	0.186	-0.772	-1.906	0.363
30	-4.288	-8.981	0.405	-1.400	-3.366	0.567
40				-2.403	-5.617	0.810
50 (walking → sedentary behaviour)				-4.634	-10.462	1.194
Sedentary behaviour – HIPA						
-8 (sedentary behaviour → HIPA)				-1.210	-1.669	-0.750
-6				-0.964	-1.332	-0.596
-4				-0.688	-0.952	-0.424
-2	-0.182	-0.582	0.218	-0.372	-0.515	-0.229
-1	-0.104	-0.333	0.125	-0.194	-0.269	-0.119
0 (reference composition)	0.000	0.000	0.000	0.000	0.000	0.000
1	0.158	-0.191	0.506	0.214	0.131	0.297
2	0.498	-0.608	1.604	0.453	0.277	0.629
4				1.037	0.632	1.443
6				1.870	1.133	2.607
8 (HIPA → sedentary behaviour)				3.365	2.028	4.703

Due to missing values in some covariates, 707 observations were included in the adjusted model.
CI, confidence interval
mm Hg, mm of mercury
HIPA, high-intensity physical activity (sum of climbing stairs [up/down], running, cycling, and rowing)
Reallocations were done relative to the reference composition (i.e., geometric mean): 380.7 and 227.9 min sedentary behaviour, 125.4 and 76.2 min standing, 48.5 and 23.4 min moving, 55.3 and 33.4 min walking, and 9.7 and 2.5 min HIPA, during leisure and work, respectively, and 457.1 min in bed.

Low-density lipoprotein cholesterol

Table C. Estimated adjusted differences in low-density lipoprotein cholesterol given time reallocations between sedentary behaviour and walking and sedentary behaviour and high intensity physical activity during leisure and work among 804 adults in the fifth examination of the Copenhagen City Heart Study

**Reallocation (min)**	**Work Estimated difference in mm Hg w. 95% CI**			**Leisure Estimated difference in mm Hg w. 95% CI**		
Sedentary behaviour – walking	**Estimate**	**Lower**	**Upper**	**Estimate**	**Lower**	**Upper**
-50 (sedentary behaviour → walking)				0.109	-0.061	0.280
-40				0.093	-0.050	0.236
-30	0.023	-0.114	0.159	0.074	-0.039	0.188
-20	0.016	-0.083	0.116	0.053	-0.027	0.134
-10	0.009	-0.047	0.064	0.029	-0.014	0.072
0 (reference composition)	0.000	0.000	0.000	0.000	0.000	0.000
10	-0.011	-0.086	0.063	-0.035	-0.086	0.016
20	-0.028	-0.218	0.163	-0.079	-0.194	0.035
30	-0.066	-0.539	0.408	-0.139	-0.337	0.060
40				-0.230	-0.554	0.095
50 (walking → sedentary behaviour)				-0.423	-1.011	0.165
Sedentary behaviour – HIPA						
-8 (sedentary behaviour → HIPA)				-0.049	-0.095	-0.002
-6				-0.039	-0.076	-0.002
-4				-0.028	-0.055	-0.001
-2	-0.015	-0.055	0.025	-0.015	-0.030	-0.001
-1	-0.009	-0.032	0.014	-0.008	-0.015	0.000
0 (reference composition)	0.000	0.000	0.000	0.000	0.000	0.000
1	0.013	-0.022	0.048	0.009	0.000	0.017
2	0.042	-0.069	0.154	0.019	0.001	0.036
4				0.043	0.002	0.084
6				0.077	0.003	0.152
8 (HIPA → sedentary behaviour)				0.140	0.005	0.275

Due to missing values in some covariates, 707 observations were included in the adjusted model.
CI, confidence interval
mm Hg, mm of mercury
HIPA, high-intensity physical activity (sum of climbing stairs [up/down], running, cycling, and rowing)
Reallocations were done relative to the reference composition (i.e., geometric mean): 380.7 and 227.9 min sedentary behaviour, 125.4 and 76.2 min standing, 48.5 and 23.4 min moving, 55.3 and 33.4 min walking, and 9.7 and 2.5 min HIPA, during leisure and work, respectively, and 457.1 min in bed.

Only individuals using antihypertensives, diuretics, or cholesterol lowering drugs

The following sensitivity analyses are conducted among individuals using antihypertensives, diuretics, or cholesterol lowering drugs only.

Systolic blood pressure

Table D Estimated adjusted differences in systolic blood pressure given time reallocations between sedentary behaviour and walking and sedentary behaviour and high intensity physical activity during leisure and work among 146 adults in the fifth examination of the Copenhagen City Heart Study

Reallocation (min)	Work Estimated difference in mm Hg w. 95% CI			Leisure Estimated difference in mm Hg w. 95% CI		
Sedentary behaviour – walking	**Estimate**	**Lower**	**Upper**	**Estimate**	**Lower**	**Upper**
-40 (sedentary behaviour → walking)				-4.948	-11.653	1.757
-30	3.562	-1.655	8.779	-3.874	-9.214	1.466
-20	2.589	-1.185	6.363	-2.714	-6.523	1.094
-10	1.431	-0.646	3.509	-1.439	-3.496	0.618
0 (reference composition)	0.000	0.000	0.000	0.000	0.000	0.000
10	-1.879	-4.583	0.825	1.682	-0.824	4.188
20	-4.626	-11.255	2.002	3.759	-1.976	9.494
30	-9.841	-23.874	4.193	6.584	-3.739	16.907
40 (walking → sedentary behaviour)				11.364	-7.088	29.817
Sedentary behaviour – HIPA						
-4 (sedentary behaviour → HIPA)				1.005	-0.752	2.762
-2	-0.344	-2.432	1.744	0.578	-0.415	1.571
-1	-0.197	-1.393	0.999	0.313	-0.220	0.846
0 (reference composition)	0.000	0.000	0.000	0.000	0.000	0.000
1	0.301	-1.521	2.124	-0.380	-1.016	0.256
2	0.964	-4.852	6.780	-0.859	-2.282	0.564
4 (HIPA → sedentary behaviour)				-2.454	-6.426	1.518

Due to missing values in some covariates, 124 observations were included in the adjusted model.
CI, confidence interval
mm Hg, mm of mercury
HIPA, high-intensity physical activity (sum of climbing stairs [up/down], running, cycling, and rowing)
Reallocations were done relative to the reference composition (i.e., geometric mean): 380.7 and 227.9 min sedentary behaviour, 125.4 and 76.2 min standing, 48.5 and 23.4 min moving, 55.3 and 33.4 min walking, and 9.7 and 2.5 min HIPA, during leisure and work, respectively, and 457.1 min in bed.

Waist circumference

Table E. Estimated adjusted differences in waist circumference given time reallocations between sedentary behaviour and walking and sedentary behaviour and high intensity physical activity during leisure and work among 146 adults in the fifth examination of the Copenhagen City Heart Study

Reallocation (min)	Work Estimated difference in mm Hg w. 95% CI			Leisure Estimated difference in mm Hg w. 95% CI		
Sedentary behaviour – walking	**Estimate**	**Lower**	**Upper**	**Estimate**	**Lower**	**Upper**
-30 (sedentary behaviour → walking)	1.093	-1.942	4.128	-0.543	-3.650	2.564
-20	0.793	-1.403	2.988	-0.355	-2.571	1.861
-10	0.437	-0.771	1.646	-0.174	-1.370	1.023
0 (reference composition)	0.000	0.000	0.000	0.000	0.000	0.000
10	-0.571	-2.144	1.002	0.165	-1.293	1.623
20	-1.402	-5.259	2.454	0.319	-3.018	3.656
30 (walking → sedentary behaviour)	-2.973	-11.138	5.191	0.457	-5.549	6.463
Sedentary behaviour – HIPA						
-4 (sedentary behaviour → HIPA)				-0.753	-1.775	0.269
-2	0.073	-1.142	1.288	-0.421	-0.998	0.157
-1	0.042	-0.654	0.737	-0.225	-0.535	0.086
0 (reference composition)	0.000	0.000	0.000	0.000	0.000	0.000
1	-0.063	-1.123	0.998	0.264	-0.106	0.634
2	-0.200	-3.584	3.184	0.587	-0.241	1.415
4 (HIPA → sedentary behaviour)				1.609	-0.702	3.920

Due to missing values in some covariates, 124 observations were included in the adjusted model.
CI, confidence interval
mm Hg, mm of mercury
HIPA, high-intensity physical activity (sum of climbing stairs [up/down], running, cycling, and rowing)
Reallocations were done relative to the reference composition (i.e., geometric mean): 380.7 and 227.9 min sedentary behaviour, 125.4 and 76.2 min standing, 48.5 and 23.4 min moving, 55.3 and 33.4 min walking, and 9.7 and 2.5 min HIPA, during leisure and work, respectively, and 457.1 min in bed.

Low-density lipoprotein cholesterol

Table F. Estimated adjusted differences in low-density lipoprotein cholesterol given time reallocations between sedentary behaviour and walking and sedentary behaviour and high intensity physical activity during leisure and work among 146 adults in the fifth examination of the Copenhagen City Heart Study

Reallocation (min)	Work Estimated difference in mm Hg w. 95% CI			Leisure Estimated difference in mm Hg w. 95% CI		
Sedentary behaviour – walking	**Estimate**	**Lower**	**Upper**	**Estimate**	**Lower**	**Upper**
-30 (sedentary behaviour → walking)	-0.192	-0.550	0.166	-0.218	-0.585	0.148
-20	-0.140	-0.399	0.119	-0.157	-0.418	0.105
-10	-0.077	-0.220	0.065	-0.085	-0.226	0.056
0 (reference composition)	0.000	0.000	0.000	0.000	0.000	0.000
10	0.101	-0.084	0.287	0.105	-0.067	0.277
20	0.250	-0.205	0.705	0.242	-0.152	0.635
30 (walking → sedentary behaviour)	0.532	-0.431	1.495	0.438	-0.270	1.146
Sedentary behaviour – HIPA						
-4 (sedentary behaviour → HIPA)				-0.042	-0.162	0.079
-2	0.017	-0.126	0.161	-0.024	-0.092	0.045
-1	0.010	-0.072	0.092	-0.013	-0.049	0.024
0 (reference composition)	0.000	0.000	0.000	0.000	0.000	0.000
1	-0.015	-0.140	0.110	0.015	-0.029	0.059
2	-0.049	-0.448	0.350	0.034	-0.064	0.131
4 (HIPA → sedentary behaviour)				0.095	-0.178	0.367

Due to missing values in some covariates, 124 observations were included in the adjusted model.
CI, confidence interval
mm Hg, mm of mercury
HIPA, high-intensity physical activity (sum of climbing stairs [up/down], running, cycling, and rowing)
Reallocations were done relative to the reference composition (i.e., geometric mean): 380.7 and 227.9 min sedentary behaviour, 125.4 and 76.2 min standing, 48.5 and 23.4 min moving, 55.3 and 33.4 min walking, and 9.7 and 2.5 min HIPA, during leisure and work, respectively, and 457.1 min in bed.
