# Supplementary material for: The physical activity health paradox and risk factors for cardiovascular disease: A cross-sectional compositional data analysis in the Copenhagen City Heart Study
Source: PLoS One. 2022 Apr 21;17(4):e0267427. doi: 10.1371/journal.pone.0267427 (PMC9022831; doi:10.1371/journal.pone.0267427)
Supplement: Attachment — Submitted filename: 210131 Review PONE-D-21-00560.docx [file pone.0267427.s009.docx]

RE: MS PONE-D-21-00560 The physical activity health paradox and risk factors for cardiovascular disease: a cross-sectional compositional data analysis in the Copenhagen City Heart Study

Review, January 28, 2021

**General Comments:**

1) This study of differential associations between domain-specific leisure time and occupational physical activity with three common cardiovascular disease risk factors addresses an important occupational and public health issue: identification of potentially modifiable underlying mechanisms of the emerging physical activity health paradox using innovative physical activity exposure assessment (wearable sensors employing accelerometry), appropriate differentiation of work and leisure, and innovative analytic approaches estimating effects using compositional data analysis. This is a seminal contribution to the evolving literature regarding the PA health paradox and deserves publication in a high-quality journal.

2) Additional major strengths of this manuscript include an obvious command over the most relevant literature in this field and appropriate citations throughout. Only few modifications are recommended (see details below). The paper is very well written, concise, and in virtually flawless English language. Provision of additional details in supplemental files are also noted as a positive feature.

3) The major acknowledged limitations include the cross-sectional design and potential sample selection bias. There are several other limitations that deserve to be discussed: conservative biases leading to underestimation of health effects due to exposure misclassification, healthy worker effects, and exclusion of eligible participants with pre-existing health conditions (such as IHD or hypertension) that are known to modify the health effects of both leisure and occupational physical activity.

4) Since data on pre-existing conditions appear to be available (these data have been used to exclude up to 2/3 of study participants) respective subgroup analyses appear to be possible within the available data set. This reviewer strongly recommends to provide additional sensitivity analyses using a larger sample that does not exclude these eligible participants and to also supplement stratified analyses in respective subsamples based on common pre-existing conditions such as IHD and hypertension that have been shown to be effect modifiers in earlier research.

5) Use consistent terminology: The title refers correctly to the technical term “physical activity health paradox,” however abstract and text often instead use “physical behavior.” I would recommend to stay consistent with the title throughout the manuscript and also with the decades-old research literature and consistently use the term “physical activity.”

n cardiovascular research the term “behaviour” is considered to point to activities over which the individual has control and thus is responsible for and able to “change behavior.” While this label has been applied in CVD research to smoking, drinking, and also leisure time physical activity, it has the tendency to distract from the social determinants of even the most private individual behaviors, it but it definitely not a good choice to characterize occupational physical activity where the activity is determined by the physical environment, explicit employer direction (e.g. the mandate to stand when serving customers in a bank even if the job could be performed sitting), or inherent in the job task itself and thus to a large extent out of the control of the individual worker. These may be subtle distinctions, however, given the long history of occupational medicine where inherently unsafe working conditions were ascribed to random acts of nature (“accidents”) or individual personal worker traits or behaviors (“accident-prone worker”, “unsafe behavior”) in order to abdicate employer responsibility and liability, shifting the more neutral term (with regard to agency/control) of “activity” to “behavior” may be perceived as implicitly “blaming the victim” – a historical legacy burden in occupational medicine and even public health that had many disastrous consequences for legions of workers, their families, and their communities in the past centuries and even today.

**Detailed Comments:**

**Abstract:**

1) Line 32: Use consistent terminology: The title refers correctly to the technical term “physical activity health paradox,” however abstract and text often instead use “physical behavior.” I would recommend to stay consistent with the title throughout the manuscript and also with the decades-old research literature and consistently use the term physical activity.

Aside: Additional comment addressing the broader research context: It is important to note that in cardiovascular research the term “behaviour” is used to indicate activities over which the individual has control and thus is in general considered responsible for and assumed to have the ability to “change behavior.” In medical and epidemiological cardiovascular disease research this label has been applied consistently to smoking, drinking, and also leisure time physical activity, under the heading “health behaviors”. While this labeling has the unfortunate tendency to distract from the social determinants (that have been identified for even the most private individual behaviors such as suicide, see Durkheim’s 19^th^ century seminal study of the same title), this is a widely accepted convention. However, to characterize OPA as a “behavior” is a problematic choice because the physical activity at work is mostly not a choice but instead determined by the physical and organizational work environment, explicit employer direction (e.g. the mandate to stand when serving customers if the job could be performed sitting), or inherent in the job task itself. Thus the intensity of OPA, its duration, and the work/rest/cycle is effectively out of the control of the individual worker, especially those who are performing high levels of OPA or repetitive tasks. Accordingly, the occupational health literature has been referred to PA at work as “occupational physical activity”, “physical workload”, “physical job demands” as more appropriate terms for most paid labor than terms that have connotations of individual choice or even a moral undertone like in “good or bad behavior.”

These are subtle distinctions, however, OPA happens in social context where performing heavy work is associated with low status, low pay, excessive health and mortality risks. Behavioralism, “the advocacy or adherence to a behavioral approach to social phenomena” as defined in the dictionary combined with a tragic history of little attention by academia, and a long and shameful history of occupational medicine where unsafe working conditions, safety hazards inherent in a specific job design or work task have been attributed to random acts of nature (“accidents” instead of “work injury”) or individual personal worker traits (“accident-prone worker”), or behaviors (“unsafe behavior”, “worker negligence,” “human factor,”) or even a kind of mental illness (“accident neurosis” “Unfallneurose,” “pension neurosis,” “Rentenneurose”) if the victim of a work-injury demands compensation - these “behaviors” (?!) of medical professionals, academicians, scientists, and legal experts ignore the root causes of work-related injuries and illnesses and collude with regulators’ and/or employers’ attempts to deny their responsibilities for providing a safe work environment and their legal liability to compensate their injured workers for lost income, health, limbs, or life. Shifting the more neutral (with regard to agency/control) term of “activity” to “behavior” (with a connotation of worker choice and good/bad or health/unhealthy behavior) may thus be a subtle form of shifting the burden of work-related injury, illness, and disability or death and the legal mandate for primary prevention at the workplace onto the individual worker and thus implicitly “blaming the victim” – a historical legacy burden in occupational medicine and even public health that had many tragic consequences for legions of workers, their families, and their communities in the past centuries and even today.

**Background:**

2) Line 71-74: The way references are inserted in this summary of the literature is confusing. The 2018 meta-analyses by Coenen et al. is cited as if it represents and individual study and for providing evidence for “beneficial health effects” and “no association with all-cause mortality” while this is a recent review that did not investigate health effects but only all-cause mortality (which is a different outcome) and actually concluded in the abstract: “**Conclusions** The results of this review indicate detrimental health consequences associated with high level occupational physical activity in men, even when adjusting for relevant factors (such as leisure time physical activity). These findings suggest that research and physical activity guidelines may differentiate between occupational and leisure time physical activity.” I would recommend to rewrite this summary, clearly differentiating between CVD risk factors, and CVD/IHD incidence, and cardiovascular and all-cause mortality, between reviews and individual studies, and between older reviews and newer reviews, because you are referring to “currently inconclusive” results.

Results regarding traditional CVD risk factors may be more inconclusive, results regarding all-cause mortality are more conclusive, at least for men. There is also a development in the literature: reviews of more recent studies and of higher methodological quality conclude that OPA effects on different outcomes are detrimental (Li 2013, Coenen 2018).

3) Line 81-82: This paragraph is clearly written, however, the last sentence starts with “therefore” but is not clearly stated why we should investigate “how,” by which mechanism, OPA affects health outcomes. (To understand it better and/or to identify additional points of interventions along the causal chain leading form OPA to health outcomes? Or to confirm biological pathways and plausibility? Or any other good reason you want to emphasize?)

4) line 90: “importantly” twice?

**Methods:**

5) line 179-190: I think this is a clear and important strength of this study: using two accelerometers and advanced algorithms that can differentiate between these different activities (with the possible exception of climbing stairs). What are the respective values of sensitivity and specificity for biking?

6) line 209-214: Eligibility criteria: Exclusion of individuals using anti-hypertensives, diuretics, or cholesterol-lowering drugs is problematic for several reasons: (1) it excludes a large percent of the study population and thereby further limiting representativeness of an already highly selected sample. (2) Any exclusion based on these medications need to be justified for each outcome separately. Does it make sense to exclude anti-cholesterol meds when examining SBP outcome, or anti-cholesterol drugs when examining WC? In the former case, one may have not have excluded people with hypertension (if this was intended) but rather those who were not diagnosed or did not seek or get or adhere to treatment… etc. these choices are likely to introduce different selection biases that need to be addressed, if not here, at least in the discussion section. (3) Most importantly, this will systematically exclude persons with some common pre-existing cardiovascular health conditions (treated hypertension and some other treated CVD) which have been shown to strongly interact with OPA in previous epidemiological research (see for example large differences in HRs in Table 4 in the Hall 2019 study you cite, and 4 other studies cited the method section of this paper justifying this approach). Did you collect data on persons with those conditions? The results section and Figure 1 seem to imply this. In fact, these exclusions lead to a loss of nearly 70% participants (1367 out of 2009 participants) due to a combination of these exclusion criteria and one unrelated factor (minimum wear time of sensors). Given these large numbers, it is important to breakdown the n for each exclusion criterion and add this in Figure 1 and/or text.

7) ibid: On the other hand, given the large sample of persons with these conditions, possibly the majority of the original study population, I would strongly suggest to examine the potential of selection bias by performing respective sensitivity analyses stratifying by these conditions and also examine how overall results would change if you included these people in the main analysis with all participants who participated and who were otherwise eligible. This is not only important for assessing quantitatively the potential risk of selection bias but even more important because 21^st^ century working populations in developed countries typically include a large percentage of workers with such conditions or medications and we need to understand if those workers experience different PA health effects in order to develop safe PA recommendations that not only differentiate between OPA and LTPA but also between healthy workers and the increasing number of workers with those conditions and in order to tailor any recommendations and interventions accordingly.

8) Line 216-223: The descriptors for different PA composites used in the text do not correspond to the answer categories shown in Supplemental Table A1 for OPA and LTPA questions (e.g. I cannot find the item “climbing stairs” in that table). Since this is part of your key exposure variables, please provide a detailed account of all related questionnaire items and the specific re-coding or combinations you used.

9) line 226: Since BP is your key outcome measure, you should provide more detail about its measurement, specifically, if your protocols adhered to any of the standard guidelines for blood pressure measurements.

10) line 275: Not sure what you mean by “results for the daily physical behavior composition as a whole” – please show and explain data in your response to this review and/or as supporting information in the appendix

11) line 283-286: “One-to-one-reallocations” separately within work and within leisure seems to be very appropriate and actual crucial for your study aims and appropriate for future interventions since both domains are highly separated in terms of degree of self-determination and require different intervention strategies. This step has helped me to re-evaluate the promise of composition analysis in this field, in early formulations I saw, this was not emphasized or I missed it. Have your PA-intensity and domain-specific approaches used by others or have most other researchers reallocated PA across intensity levels and across domains? It may be worth highlighting your approach if it is innovative as such here (providing the rationale) and in the discussion (comparing with others and pointing to consequences), as this seem to me a major contribution of your paper.

12) I am pleased to see that this study defines “sedentary behavior” at work as sitting and does not include light standing/walking work because the common practice of many researchers combining sitting and “light standing” work into a common category “sedentary” is problematic because there is strong evidence that standing alone (with some walking but an mostly upright work posture) is a potentially strong risk factor for progression of atherosclerosis and CVD and mortality compared to sitting (even after adjustment for SES and other potential confounders (see Canadian study by Peter Smith, 2018 on AMI, and several Finnish studies by Krause et al. and Hall 2019 whom you cite). Combining sitting with standing causes a strong conservative misclassification bias by inflating the baseline risk in any reference group that contains standing work in addition to sitting work. Question: Could you assess the magnitude of this potential misclassification bias by a (not recommended) sensitivity analysis that uses “sitting or standing at work” (without lifting) as the reference group? That would be a nice extra contribution to the field and could be put into the appendix for a reference for other researchers. Your study, with objective measurements would be uniquely able to compare reference groups based on sitting alone or sitting and standing combined.

Results:

13) line 307 ff and Table 1: The text and tables describe the distribution of study characteristics as medians and the first and third quartile. Is there any specific reason for this choice? Regardless, while these descriptors are not wrong and could still be provided in the appendix, this table should show the means the full range of all continuous variables to allow the reader to better compare this study population and its exposures and other covariates with other study populations in the literature that typically show means and full ranges.

14) line 345-49 Results for SBP: The description of results focuses on the patterns of point estimates of change in terms of the direction of change, which are the most important results. However, the rest of the description ignores the second most important data: effect sizes and instead implicitly focuses on statistical significance testing (using CIs as a substitute for p-values) while ignoring additional substantive quantitative information contained in both point estimates and CIs.

For example, reallocation from reference to more sedentary behavior shows only small increases in BP between +0.21 and +0.60 mmHg for LTPA but rather substantial decreases from -0.95 to -6.66 mmHG for OPA. For a 30 min reallocation of walking to sedentary behavior, the absolute effect size (absolute change in BP) for OPA is 11 times larger than for LTPA: -6.66 compared to +0.6 mmHg and reaches an effect size that is substantial and could be expected to decrease the risk of CVD by over 20% based on the known linear relationship between SBP and CVD.

Although it is correct that all CIs include zero, there are notable differences in CIs across domains: CIs for LTPA point estimates are more balanced around the zero value while CIs for OPA point estimates are heavily tipped towards negative values (decreases) in BP. For the 30 min reallocation of walking to sedentary behavior mentioned above, the CIs for LTPA covers values between -2.66 to +3.85 and for OPA -16.19 to +2.88. These CIs are wide and thus indicate relative imprecise estimates that include zero, therefore the data are compatible with effects in either direction, especially for LTPA: the range of values within the CI interval for LTPA are nearly equally compatible with similar decreases or increases in SBP. In contrast, the majority of values within the CI for OPA are negative and negative values much larger than positive values, the data are therefore much more compatible with large decreases in SBP than (much smaller) increases in SBP. Taking these more detailed observations together, the overall results are clearly much more compatible with a detrimental effect of walking at work than not and also point in this direction much more than the results point into the direction of the smaller potential beneficial effect of LTPA. Despite a lack of statistical significance, these results are much more compatible with your hypothesis of the presence of the PA health paradox than with similar effects across domains. While your description of the results is not wrong, it implicitly relies too much on the single data point of statistical significance. Your description and especially your overall interpretation of the data should consider the entirety of information contained in CIs, not just the equivalence of one specific data point at the edge of the 95% CI interval that is equivalent to a p-value of 0.05. Instead of answering the narrow question, do 95% of all values lie above this one arbitrary point along the continuum of results within the CI, one needs to consider if the majority of points within the CI points to an increase or decrease in SBP. Several decades of scholarly work, textbooks of modern epidemiology (e.g. by Rothman, Greenland et al), and official statements of the American Statistical Association (2015) all encourage to base conclusions on effect estimation and making full use of the data included in CIs instead of relying primarily on statistical significance testing or its equivalent in the interpretation of CIs.

In this spirit, I would suggest to represent all effect estimates in the tables with the same font and not to bold those data and CIs that do not include zero. The emphasis should be on results that are substantitive, e.g. point to a relevant change in the health risks that are known to be associated with your cardiovascular risk factor under study. For example, it is known that above about 115 mmHG, the relationship between SBP and CVD outcomes is monotone positive and virtually linear and that the risk of acute myocardial infarction (AMI) in a population increases by about 10% for each 2-3 mm Hg average increase in any population. Since AMI is a rather common disease, a 10% increase means thousands of people in DK and millions of people worldwide. Therefore, even a “small” 1 or 2 mmHG change in SPB that may be deemed irrelevant by clinicians for an individual patient is considered important in occupational and public health concerned with prevention of CVD in whole populations. From that perspective, researchers have considered even relative relative small changes of 1 mmHG or less as substantive and of public health significance. From that perspective, it is much more important to ask the question if 95% CI’s include values of that magnitude or more than the question if it includes zero values.

15) line 350-54 Results for WC: This description mentions results of “less sedentary behavior and more walking,” but not of “more sedentary behaviour and less walking” although the latter reallocation leads to substantially larger BP changes for both LTP and OPA. Similarly to SPB above, reallocation of 30 min of occupational walking to sedentary work led to a very substantial -5cm reduction of WC, five times as much than the reduction observed when the same amount of walking during leisure is reallocated to sedentary behavior.

16) line 354-55 WC: the sentence “We found no association during work” is not backed up by the data. I would suggest to replace by a similar wording you used for HIPA and LDL in the next paragraph: “During work, the estimates followed the same pattern, but were even smaller …”

17) line 357-362 Results for LDL, Table 4, row -1 min, column Work: CI does not include point estimate (data entry error?)

**Discussion:**

18) line 377-383 SBP: You state correctly “Although not statistically significant, these findings support…” the PA paradox. Since SBP is one of the most important global CVD risk factor, you may want to provide the reader with a more detailed interpretation of results that help the reader to understand why you do not dismiss results that are not statistically significant. See comment #14 above.

19) line 391: replace “makes” by “make” (plural)

20) line 390-396: suggest to add the reference 18 to reference 14 each time mentioned here and add also consider to reference a couple of review papers that summarize the effect of BP and HR on CVD (some are cited in reference 18) if you have enough room for references. You may also mention the very simple explanation that cumulative exposure to higher BP and HR during work hours can be expected to increase CVD risk based on these positive associations between SPB and HR – it is a predictable outcome based on the well-established hemodynamic theory of arteriosclerosis (review paper by S Glagov 1988 “Hemodynamics and atherosclerosis” and/or M.J. Thubricar, Vascular mechanics and pathology, Springer, New York, 2007)

21) line 401: insert “objective” before “device-based”

22) line 426-33: In this paragraph you mention replacing sedentary activities with more walking at work may increase SBP by 1.7 mmHg and how this represents a substantially increased CVD risk on a population level. This makes totally sense and is in line with my earlier comments asking for stating this clearly. However, I think it is crucial to also point out the even higher prevention potential for replacing walking time at work by sedentary time by stating something like that (see comment #14 above) “While reallocation from reference to more sedentary LTPA behavior shows only small increases in BP between +0.21 and +0.60 mmHg, reduction of walking at work by 30 min and allocating this time to sitting at work results in rather substantial average decreases -6.66 mmHG. For a 30 min reallocation of walking to sedentary behavior, the absolute effect size (absolute change in BP) for OPA is infect 11 times larger than for LTPA: -6.66 compared to +0.6 mmHg and reaches an effect size that is substantial and could be expected to decrease the risk of CVD by over 20% based on the known linear relationship between SBP and CVD.” I think it is important to state this observation explicitly because the average reader who is has been reached by public health messages advocating a decrease of sitting at work will not pick this counter-intuitive statement that it may be actually much more advantageous to replace walking by sitting at work.

23) line 450-52: suggest to replace “total energy expenditure” by “diet” here because (a) energy expenditure itself is an independent CVD risk factor and (b) an integral part of relative aerobic workload the OPA measure that takes the important mismatch between physical work demands in terms of energy burned and workers cardiorespiratory fitness i.e. the capacity to burn that energy (V0_2_max) into account. (c) There is little evidence that total energy expenditure is most important for WC or obesity, it seems that the source of energy (sugar vs protein or fat) and endocrine effects of sugar and thus the high sugar content of soda-drinks and processed food may instead be the determining factors for central obesity (see comprehensive in-depth review in book by Gary Taubes for the specific literature: “Good calories, bad calories: fats, carbs, and the controversial science of diet and health”).

24) line 499-500: This statement (“i.e. only borderline during work”) is vague because it does not explain what “borderline” it refers to although I would assume that it is based on comparisons of statistical significance between PA domains instead of any minimum relevant effect size or effect estimation. The statement is problematic because the actually measured effects are different than this statement suggests: 50% higher for LTPA compared to OPA when adding 2 min of HIPA (0.02 vs 0.01 mmol/l), identical when adding or substracting 1 minute HIPA (0.01 mmol/l) and 50% higher for OPA (0.03 vs 0.2 mmol/l) when reducing HIPA by 2 min as shown in Table 4. Pointing out that only the LTPA findings are statistically significant does not summarize these results well. It looks more like consistent but small substantial effects overall regardless of direction and statistically significance testing. However, this assessment would be premature without consideration of how important any small LDL changes in the observed range maybe at the population level. You provide this info in your next paragraph: 30% for 1 mmol; this translates into 0.3% -0.9% lower IHD mortality, this seems to be still substantial on a population level although is much smaller than the examples given for SBP above. Your conclusion that the relationship between PA domains and LDL-C is unclear holds

However, this needs to be balanced against the potentially larger detrimental effects these reallocations may have with regard to SBP.

25) line 500 cited reference 47 by Honda 2014: It may be interesting to note that Honda reported (under fully adjusted model 3 in their Table 3) that 60 min of sedentary LTPA increases LDL by 0.77 mmol/L, while sedentary OPA, in contrast, decreases LDL by the -0.73 mmol/l. Similar paradoxical effects were shown for SBP (and less convincingly for WC) although the authors choose to totally ignore these findings because they were not statistically significant. However, a closer examination of confidence intervals (-1.78 to 0.31 for LDL) shows that their data are much more compatible with the PA paradox than not. I attach this table with my highlights here for your quick reference.

26) Line 510: “being overweight” is entered as a CVD risk factor. May be replacing it by “obesity” would be appropriate: If I recall it correctly, a closer look at the evidence suggests a bi-modal risk relationship between BMI with elevated risks for underweight and normal weight persons, lowest risk for overweight, and increasing risk for obese and above.

Aside: Interestingly, in several Finnish cohort studies, BMI was not at all (HR=1.0) related to CVD and mortality outcomes in models that include occupational physical activity (and virtually all other known CVD risk factors). Hi BMI is also partly a function of high muscle mass which is related to the capacity to perform heavy labor and thus may be an intricate correlate of high OPA but with no independent effect on CVD once OPA is controlled for. Consequently, the literature on overweight and CVD is probably still inconclusive.

27) Line 519-20: From a primary prevention perspective it is paramount not to exclude 2/3 of the general population that has these potentially modifying factors – we want to know how this works out in real populations not a selected minority group of mostly healthy individuals who are already at the lowest risk for CVD, they are the least in need of additional research!

It seems that you have data on persons with theses potentially confounding or effect modifying factors (based on the many exclusions you reported based on these factors). Could you rerun your analyses with everybody included and compare this with your current results as a sensitivity analysis? And thereafter investigate this further with stratified analyses by how much these factors may change relationships (in a supplement or in a separate paper)? See also comments and specific suggestions in comments #6 and #7 above.

28) Line 527-529: While I agree that representativeness is not as important as validity (because what sense would it make to generalize findings of questionable validity?). I also agree that normal physiology needs to be studied in normal or healthy persons (but not necessarily young, or college-educated, or athletic etc. because what is normal will change during the life cycle – think of menopause and other normal changes). “Normal” is a quite difficult selection criterion as is “external”, after there are not really any people on earth that are not influenced by a myriad of “external” factors, medication is just one of the ones we actually can and should measure and thus take into account.

Instead of controlling for these factors by either excluding participants or by adjustment in multivariate models, these factors and their influence on the exposure-disease relationships need to be actively investigated. Two basic steps have been suggested above: investigate the potential impact quantitatively by comparing changes in effect estimates (not statistical significance!) caused by adding this modifying factor to multivariate models and investigate multiplicative and additive interactions or (better) by comparing effect estimates from analyses stratified on categories of this potentially modifying factor. (More sophisticated mediation analysis may be needed to disentangle the different effects those factors may have but they are best reserved for prospective data with repeat measures).

Exclusion of people with potentially effect modifying characteristics is not helping to clarify their potential impact and is actually detrimental for primary prevention efforts that need to address high risk populations that are often characterized by these very factors that led to the exclusion of otherwise eligible study participants. This is the main weakness of this paper but it could be addressed by adding the suggested sensitivity analyses.

The selection of analytic samples from general study populations for epidemiological investigation into the associations between ubiquitous PA exposures and highly prevalent CVD risk factors with a goal to solve occupational and public health problems associated with these risks, need to be different than samples selected for the study of physiological experiments, or sports performance, or clinical interventions designed for patients only. While I think that your study is a wonderful contribution, it would be best to strive for resolving these issues instead of excluding two thirds of eligible participants from further investigation (and thus basic knowledge needed to design interventions for them). I hope it is clear that I am not asking for restricting research to representative samples, I agree wholeheartedly with Rothman whom you cite on this topic, but he and I myself actually argue for non-representative analytic sampling strategies, including stratified analyses, that allow to make inferences of potentially modifying factors such as pre-existing CVD that have already been shown to exert strong modifying effects on CVD risk in other studies and are common in our societies and our aging workforce too. Again, sampling for such a study may include oversampling certain subgroups to make comparisons between subgroups more comparable (study design B in Rothman 2012 you cited). Thanks for citing his papers, it was a pleasure to reread them!

29) line 537: limitations of exposure assessment by accelerator data: You may want to mention three additional important limitations here: 1) inability to consider the weight of materials, people, or tools handled (need for additional ergonomic assassments), 2) questionable implicit assumption that observed exposure during a short time window accurately reflects typical exposure (need to assess that separately by diary or survey or experts), 3) inability to assess past and cumulative overall exposure that may be most relevant for adverse chronic health effects such as CVD (need for repeat measurements over long study periods that is typically not feasible; need for assessing past exposure through detailed job histories based on self-report, administrative records, and expert assessments, e.g. a job exposure matrix, JEM). Occupational exposure assessment that solely relies on accelerometer-based snap-shots of current exposure is not necessarily more valid than self-reports or JEMs, and may in fact lead to massive exposure misclassification, especially if job tenure is short which is the case in many low SES jobs.

30) line 548-51 LDL-C: Comment: LDL-C appears to be indeed a strong and, as prospective cohort studies of OPA and CVD have shown, also a rather independent CVD risk factor that is therefore not a prime candidate for being a major mediator or confounder of the OPA -CVD relationship. However, this needs to be determined in prospective studies that also take the effects of widely-prescribed lipid-lowering drugs and pre-existing CVD into consideration.

31) line 566-67: See comment # on “borderline” Although not the subject of any academic paper I am aware of, it is well-known among occupational health researchers, ergonomists, and manual workers themselves, that physically demanding work is only endurable if workers avoid as much as possible HIPAs that make them sweat or breathless during work (i.e. avoid the intensity that may produce training benefits in terms of cardiorespiratory fitness as it does during LTPA or athletic training) because working conditions typically do not allow for self-paced work with adequate recovery time and it is impossible for most workers to change out of sweat-drained wet clothes into dry clothes causing during work shifts. Therefore, larger sample sizes are not likely to change the noted limitation of few minutes of HIPA, it is an inherent characteristic of most real-world heavy labor.

Getting more accurate data instead will be dependent on implementing another form of composition analysis: improvement of all sources of exposure misclassification of OPA as outlined in comment #29 above: combining sensor-based methods with detailed job histories, JEMs, and other available data (e.g., administrative cumulative data on work hours, workdays, and leave time such as vacation, sick days, family leave, continuing education or retraining, unemployment, disability, retirement etc.) to construct more accurate exposure measures, and exposure measures that actually capture the relevant cumulative OPA exposures. Such improved imprecision of exposure assessment may help to reduce the conservative misclassification bias that plagues the literature and may be responsible for imprecision, underestimation of related chronic health consequences, and an overall inconclusive evidence base. Further, avoiding of categorization of continuous outcome measures artificially introduced by researchers during survey development or during data analysis, and replacing quantile categorization by categories that are demarcated by actual threshold effects in risk, is a much more promising and more feasible and cost-efficient approach for yielding more precise and also more valid health effect estimates.

32) line 569-72: I would also reference Peter Smith’s Canadian landmark cohort study on AMI here (“The relationship between occupational standing and sitting and incident heart disease over a 12-year period in Ontario, Canada,” Am J Epidem, 2018;187:27-33), which is also good example how JEM’s can be utilized for objective OPA assessment in very large samples that are typically beyond feasibility regarding accelerometer- or direct ergonomic observation.

33) line 577: Can you be specific about the health outcomes and provide respective references here?

34) line 584: I would suggest to cite here some of the existing evidence regarding null effects of LTPA (e.g., Krause N, Brand RJ, Arah OA, Kauhanen J, Occupational physical activity and 20-year incidence of acute myocardial infarction: results from the Kuopio Ischemic Heart Disease Risk Factor Study, Scand J Work Environ Health, 2015;41(2):124-139) and regarding even detrimental effects of LTPA (e.g., Eaton CB, Medalie JH, Flocke SA, Zyzanski SJ, Yaari S, Goldbourt U,Self-reported physical activity predicts long-term coronary heart disease and all-cause mortalities. Twenty- one-year follow-up of the Israeli Ischemic Heart Disease Study. Arch Fam Med 1995;4(4):323–329; more references can be found in PA guidelines talking about risks of LTPA), as well as specific literature regarding “overtraining effects in sports”?

35) line 587: Reference 73(Korshøj et al. 2015) does not support the introduction of aerobic exercise during work. This RCT showed average increases of systolic blood pressure by 3.6 mm Hg (95% CI 1.1-6.0) relative the control group. It is important to cite this study but as a warning regarding unintended consequences of such a program instead as confirmation for this approach.

36) line 587-99: The point is not to consider OPA in PA, that is already being done implicitly by counting any PA towards recommended PA goals. The pressing issue for a revision of PA guidelines is instead to acknowledge the PA health paradox and the need for more targeted and safe PA recommendations that take potential differential effects of OPA and LTPA and baseline CVD health status into account.

37) line 596: see comment #31 above re “larger sample size”. Maybe avoidance of unnecessary misclassification is worth mentioning here?

38) line 602-609: This overall conclusion summarizes the main findings well and is backed up by the data. Given the discussion of WC and LDL_C results above, it may be appropriate to replace “No difference between domains ..” by “No consistent differences ….”
