# Supplementary material for: The physical activity health paradox and risk factors for cardiovascular disease: A cross-sectional compositional data analysis in the Copenhagen City Heart Study
Source: PLoS One. 2022 Apr 21;17(4):e0267427. doi: 10.1371/journal.pone.0267427 (PMC9022831; doi:10.1371/journal.pone.0267427)
Supplement: Attachment — Submitted filename: Response to reviewers_Johansson et al_Revision 2_070322.docx [file pone.0267427.s011.docx]

# Response to reviewers’ comments

PONE-D-21-00560
The physical activity health paradox and risk factors for cardiovascular disease: a cross-sectional compositional data analysis in the Copenhagen City Heart Study

Dear Dr. Gianluigi Savarese,

We would like to acknowledge you and the reviewer for taking your time to assess our manuscript and providing valuable feedback. Re the Journal requirements, we have checked the reference list to make sure it is complete and correct. A point-by-point response can be found below. Changes made in the manuscript have been highlighted using the track-changes function in Word.

With kind regards on behalf of all authors,

Melker S. Johansson

**Additional Editor Comments:**

Minor comments:
1. The Background is definitely too long and reads as a review. The Authors are invited to shorten this section. Most of what explained here belongs to the discussion.

Response: We acknowledge that the introduction was too long.

Action: The introduction has been shortened substantially from 636 words to 352 (p. 3-4, line: 55-84.

2. There were some missing data. How were they handled?

Response: We interpret the editor’s comment to relate to the missing values of some of the covariates included in the adjusted regression models. We acknowledge that the sentence given in the legends of Table 3 and Table 4 was not clear and have therefore paraphrased.

Action: The sentence in the legend of Table 3 and Table 4 now reads: “69 observations were not included in the adjusted models due to missing values in some covariates.” (p. 18 and 19). In addition, we have added the following sentence in the Methods section: “Observations with missing values in the covariates were not included in the adjusted models (n=69).” (p.11, line: 247-248).

**Reviewers' comments:**

**Reviewer 1**

I am willing to accept the manuscript. I will just recommend the authors to go through the paper and review carefully grammar and spelling to make sure there are no mistypes or something. Otherwise, congratulations on this good paper.

Response: We thank the reviewer for the comments, which have helped us improve the manuscript. Finally, we have reviewed the manuscript for grammatical and spelling errors and typos.
