# Supplementary material for: The physical activity health paradox and risk factors for cardiovascular disease: A cross-sectional compositional data analysis in the Copenhagen City Heart Study
Source: PLoS One. 2022 Apr 21;17(4):e0267427. doi: 10.1371/journal.pone.0267427 (PMC9022831; doi:10.1371/journal.pone.0267427)
Supplement: Attachment — Submitted filename: PONE, REVIEWER COMMENTS.docx [file pone.0267427.s008.docx]

To be accepted for publication in PLOS ONE, research articles must satisfy the following criteria:
1. The study presents the results of original research. THIS IS ORIGINAL RESEARCH
2. Results reported have not been published elsewhere. RESULTS ARE NOT PUBLISHED BEFORE
3. Experiments, statistics, and other analyses are performed to a high technical standard and are described in sufficient detail. ALL THESE ARE WELL EXPLAINED
4. Conclusions are presented in an appropriate fashion and are supported by the data. ARE STILL INSUFICIENTS, CAN BE IMPROVED
5. The article is presented in an intelligible fashion and is written in standard English. WELL DONE
6. The research meets all applicable standards for the ethics of experimentation and research integrity. YES
7. The article adheres to appropriate reporting guidelines and community standards for data availability. YES

Background

The purpose of the study needs to be fixed (talk about objectives, believe there is one)

Results

First paragraph> some data within the text are repeated in Table1, and again for table 2

Line 336> how was the judgement of the models/residuals? That nay be important to explain a little bit more

In my opinion, tables 3 and 4 are huge, and that makes a little bit harder to understand what is there.

Discussion

Major concern> Results indicated that less SB and more walking to be associated with a larger WC. This is a major finding contradicting what is known. The explanation of this finding needs to be addressed deeper making a better case. What is already explained is confusing. Occupations that involve less SB and long walking (more PA) may impact WC in a positive way independently of socioeconomic status. I would like to see more on this to be more convincing. Low income is associated with poor health, but I am not sure that low-income people who work in environments that include high PA would have larger WC. In my opinion it is important to identify other variables or circumstances that may have some associations with the previous finding to explain, convince, and make a better case.

LDL-C> same as above. These 2 results need to be deeper explained

Compensation and nutrition may have something here in both variables

Methodological considerations

LDL was chosen due to the relationship with CVD, however, HDL could be more important due to the protective effect, and also because PA seems to have incremental effects on HDL levels but less effects on LDL. In this case, it may be important to explain why HDL was not used as variable.

Perspectives

From line 572 to line 584, if compensation for PA is included, it would help to understand people’s behavior and some results from the study.

Conclusions

I would like to see the take home message here and not the already known results from the study. What is the impact of the study, what it apports to the knowledge, why is important to consider SB, walking, and HIPA during leisure and work.
